# Type I collagen secreted in white matter lesions inhibits remyelination and functional recovery

**DOI:** 10.1038/s41419-025-07633-w

**Published:** 2025-04-13

**Authors:** Reiji Yamazaki, Morio Azuma, Yasuyuki Osanai, Tom Kouki, Takeshi Inagaki, Akiyoshi Kakita, Masaki Takao, Nobuhiko Ohno

**Affiliations:** 1 Department of Anatomy, Division of Histology and Cell Biology, School of Medicine, Jichi Medical University, Tochigi, Japan; 2 Department of Pharmacology, Division of Molecular Pharmacology, School of Medicine, Jichi Medical University, Tochigi, Japan; 3 Department of Anatomy, Division of Forensic Medicine, School of Medicine, Jichi Medical University, Tochigi, Japan; 4 Department of Pathology, Brain Research Institute, Niigata University, Niigata, Japan; 5 Department of Clinical Laboratory, National Center of Neurology and Psychiatry, Tokyo, Japan; 6 Division of Ultrastructural Research, National Institute for Physiological Sciences, Okazaki, Aichi Japan

**Keywords:** Oligodendrocyte, Neuroimmunology

## Abstract

White matter injury is caused by cerebral blood flow disturbances associated with stroke and demyelinating diseases such as multiple sclerosis. Remyelination is induced spontaneously after white matter injury, but progressive multiple sclerosis and white matter stroke are usually characterised by remyelination failure. However, the mechanisms underlying impaired remyelination in lesions caused by demyelination and stroke remain unclear. In the current study, we demonstrated that collagen fibres accumulated in the demyelinated lesions of multiple sclerosis patients (age range 23–80 years) and white matter lesions of stroke patients (age range 80–87 years), suggesting that the accumulation of collagen fibres correlates with remyelination failure in these lesions. To investigate the function of collagen fibres in the white matter lesions, we generated two types of white matter injury in mice. We induced focal demyelination by lysolecithin (LPC) injection and ischemic stroke by endothelin 1 (ET1) injection into the internal capsule. We found that type I collagen fibres were secreted in ET1-induced lesions with impaired white matter regeneration in the chronic phase of disease. We also showed that monocyte-derived macrophages that infiltrated into lesions from the peripheral blood produced type I collagen after white matter injury, and that type I collagen also exacerbated microglial activation, astrogliosis, and axonal injury. Finally, we demonstrated that oligodendrocyte differentiation and remyelination were inhibited in the presence of type I collagen after LPC-induced demyelination. These results suggest that type I collagen secreted by monocyte-derived macrophages inhibited white matter regeneration, and therefore, the modulation of type I collagen metabolism might be a novel therapeutic target for white matter injury.

## Introduction

White matter is a region of the central nervous system (CNS) composed of numerous myelinated nerve fibres ensheathed by oligodendrocytes. White matter injury is caused by cerebral blood flow disturbances associated with stroke and demyelinating diseases such as multiple sclerosis (MS) [1–4]. White matter lesions are mainly identified by MRI and histological analysis [5, 6], and they cause cognitive dysfunction, depression, and motor paralysis, as well as increasing the risk of developing dementia [3, 4, 7–9]. White matter stroke and progressive MS lead to demyelination and impaired remyelination by oligodendrocytes. The induction of remyelination in white matter injury is thought to be critical for avoiding secondary axonal damage following remyelination defects [4, 9, 10], and achieving functional recovery [11–13]. Therefore, oligodendrogenesis, a process involved in remyelination, has been the focus of studies of white matter damage associated with cerebral ischaemia [14–16]. However, there is still no effective treatment for white matter damage. Reasons for the lack of effective treatments may include the presence of factors that inhibit regeneration in complex pathologies and problems with the physical environment around axons. Therefore, understanding the mechanisms underlying impaired white matter regeneration, which is commonly observed in ischaemic white matter damage and progressive MS, would be beneficial for developing treatments.

Myelin repair is necessary to guide white matter regeneration after injury, which might lead to the promotion of functional recovery. The secretion of inflammatory and anti-inflammatory factors by microglia and astrocytes was reported to be important for remyelination [17, 18]. Previous studies reported monocyte-derived macrophages (MDM) that infiltrated into lesions after blood-brain barrier breakdown were protective but also exacerbated disease, which involved phagocytosis and the secretion of interleukin-6 (IL-6) and nitric oxide during neuroinflammation [19]. Microglia contribute to remyelination by removing myelin debris but also inhibit the differentiation of oligodendrocyte precursor cells (OPC) into myelinating oligodendrocytes by secreting transforming growth factor-β, IL-1β, and tumour necrosis factor-α [20–22]. However, microglia and MDM have different gene expression profiles and morphologies [23], as well as different roles in pathological situations. Therefore, to elucidate the pathophysiology of white matter injury and develop effective interventions, it is important to understand the functions of microglia and MDM.

Recently, we reported that lysolecithin (LPC)-induced demyelination at the internal capsule (IC) resulted in acute motor deficits, followed by subsequent remyelination-associated functional recovery, whereas an endothelin 1 (ET1)-induced stroke model resulted in lasting motor deficits [24–26]. Furthermore, we found that type I collagen was present in large amounts in early-stage ET1-induced lesions compared with LPC-induced lesions [25]. A previous analysis comparing non-lesions and demyelinated lesions in autopsied brains of MS patients reported the expressions of some collagen types were upregulated, and that type I collagen was predominantly deposited around veins in lesion areas compared with non-lesion areas [27, 28]. Therefore, the extracellular matrix is thought to participate in the pathology of white matter injury. The differentiation of cultured oligodendrocytes was inhibited in the presence of fibroblasts, the main collagen-forming cells [29]; however, whether type I collagen deposition inhibits the regeneration of white matter after brain injury is unknown.

Therefore, we examined whether collagen fibres were present in the white matter lesions of biopsied brains of MS patients and postmortem brains of stroke patients. Then, we investigated the source of collagen fibres produced in the lesion that prevented remyelination. Furthermore, we clarified that MDM produce type I collagen in the white matter after ET1 injection into the IC. Finally, we demonstrated that the presence of type I collagen inhibited oligodendrocyte differentiation and impaired white matter regeneration using an IC demyelination mouse model. Our results suggest modulating collagen formation has therapeutic potential for white matter injury.

## Results

### Collagen fibres are present in the white matter lesions of MS patients and stroke patients

**Fig. 1:**
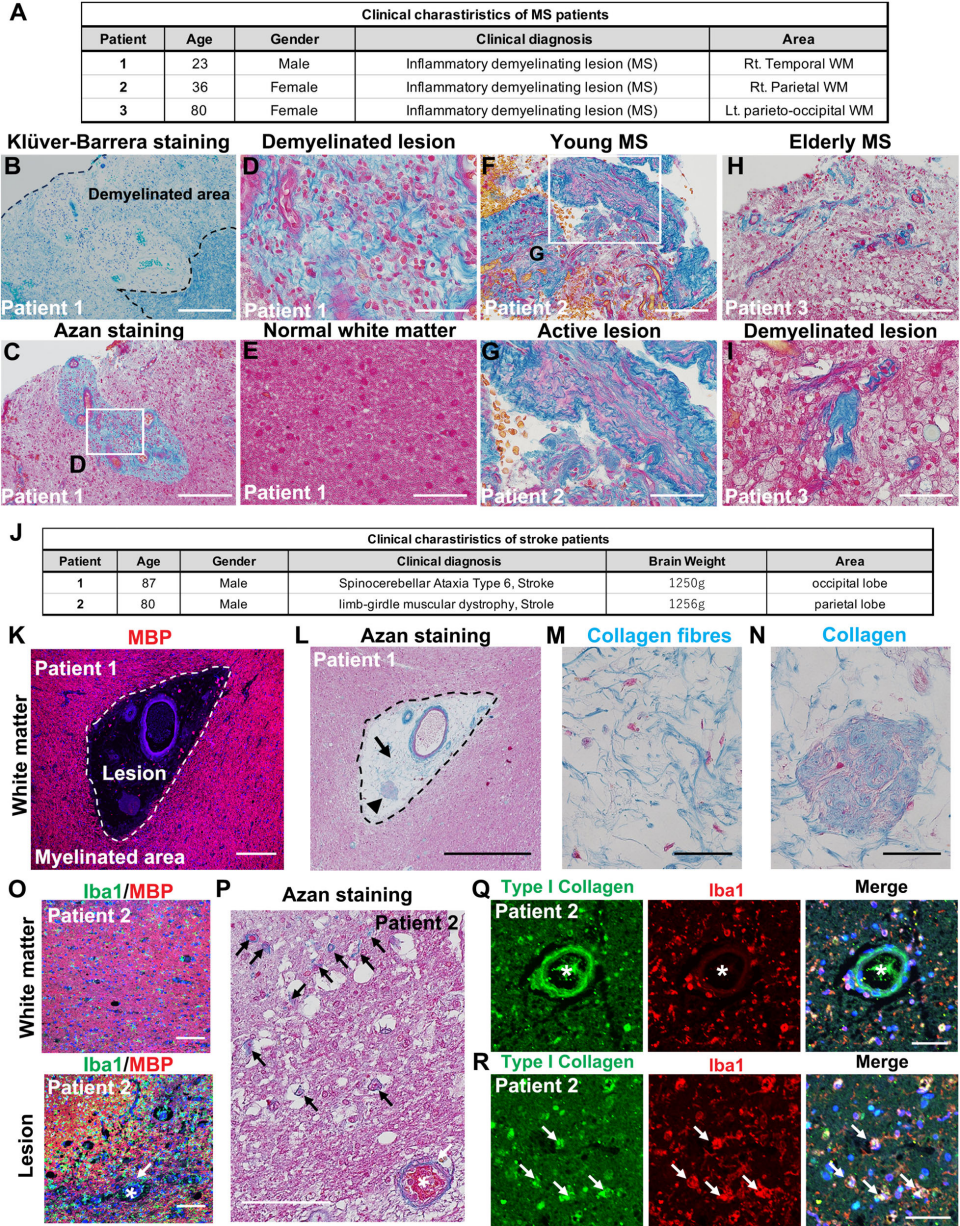
Collagen fibres accumulate in the white matter (WM) lesions of MS patients and stroke patients. **A** Clinical characteristics of MS patients. **B**, **C** Klüver-Barrera and Azan stained images of paraffin sections from MS patients. **B** The black dotted line indicates the demyelinated lesion. The blue-stained area indicates white matter with remaining myelin. Scale bar, 200 µm. **C** Collagen fibres (blue) present in active lesions of patient 1 with MS. Scale bar, 200 µm. **D**, **E** A demyelinated lesion and normal white matter of patient 1 with MS. Scale bars, 50 µm. **D** An enlarged image of the boxed area in (**C**). **F** Collagen fibres (blue) present in active lesions of patient 2 with MS. Scale bar, 100 µm. **G** An enlarged image of the boxed area in (**F**). Scale bar, 50 µm. **H, I** Collagen fibres (blue) remain in lesions of elderly patient 3 with MS. **F**–**I** All observed areas show demyelinated lesions. Scale bars, 100 (**H**) or 50 µm (**I**). **J** Clinical characteristics of stroke patients. **K** Immunofluorescence images of MBP (red) in paraffin sections from patient 1. The lesion is indicated by a white dotted line. Scale bars, 200 µm. **L** An Azan-stained image of a paraffin section from stroke patient 1. The black dotted line shows the same area as the white dotted line in (**K**). Black arrow and black arrowheads indicate the areas of (**M**, **N**), respectively. Scale bar, 500 µm. **M**, **N** Collagen fibres and aggregated collagen are present in the white matter lesion of stroke patient 1. Scale bars, 50 µm. **O** Double immunofluorescence images of Iba1 (green) and MBP (red) in paraffin sections of non-lesional white matter and in a lesion from patient 2. An asterisk and a white arrow in the lesion indicate the same blood vessel as in (**P**). Scale bars, 100 µm. **P** An Azan-stained image of a paraffin section from patient 2. Black arrows indicate collagen fibres in the lesion. Scale bar, 100 µm. **Q**, **R** Double immunofluorescence images of type I collagen (green) and Iba1 (red) in paraffin sections from patient 2. Scale bars, 50 µm. (**P**, **Q**) Asterisks indicate type I collagen-positive blood vessels. **R** Type I collagen-positive signals (green) are colocalised with Iba1 immunoreactivity (red) (white arrows). Nuclei were counterstained with Hoechst (blue).

To examine whether collagen fibres are present in the white matter lesions of humans, we performed histological analysis using brain sections of MS patients and stroke patients (Fig. 1A, J). Klüver-Barrera staining was used to evaluate demyelinated lesions (Fig. 1B, Fig. S1). Demyelinated lesions (black dotted area) and white matter with remaining myelin (blue stained areas) were observed (Fig. 1B). We identified demyelinated areas according to Klüver-Barrera staining, and white matter lesions were observed in all patients (Fig. 1B and Fig. S1). Next, we examined biopsied brain sections from three patients diagnosed with MS by Azan staining, which detects collagen fibres (blue), nuclei (red), and cytoplasm (red). Collagen fibres were observed in the active lesions of a young MS patient 1 (23 years old) and patient 2 (36 years old) (Fig. 1C, D, F, G), but not in normal white matter (Fig. 1E). Collagen fibres were present in lesions of an elderly MS patient 3 (80 years old) (Fig. 1H, I). Next, we examined the white matter of a brain section from stroke patient 1 (87 years old) (Fig. 1K), and found many collagen fibres and aggregated collagen had accumulated in the white matter lesions (Fig. 1L-N). Iba1 and myelin basic protein (MBP) staining indicated white matter lesions compared with non-lesional white matter in stroke patient 2 (80 years old) (Fig. 1O). Collagen fibres were also detected in the white matter lesions (Fig. 1P). Type I collagen was present and colocalised with Iba1-positive microglia/macrophages in lesions of the stroke patient (Fig. 1Q, R). These analyses indicated that collagen fibres were produced in the active lesions of biopsied brains from MS patients and the lesions of white matter stroke patients.

### Collagen fibres accumulate in chronic lesions after white matter injury

**Fig. 2:**
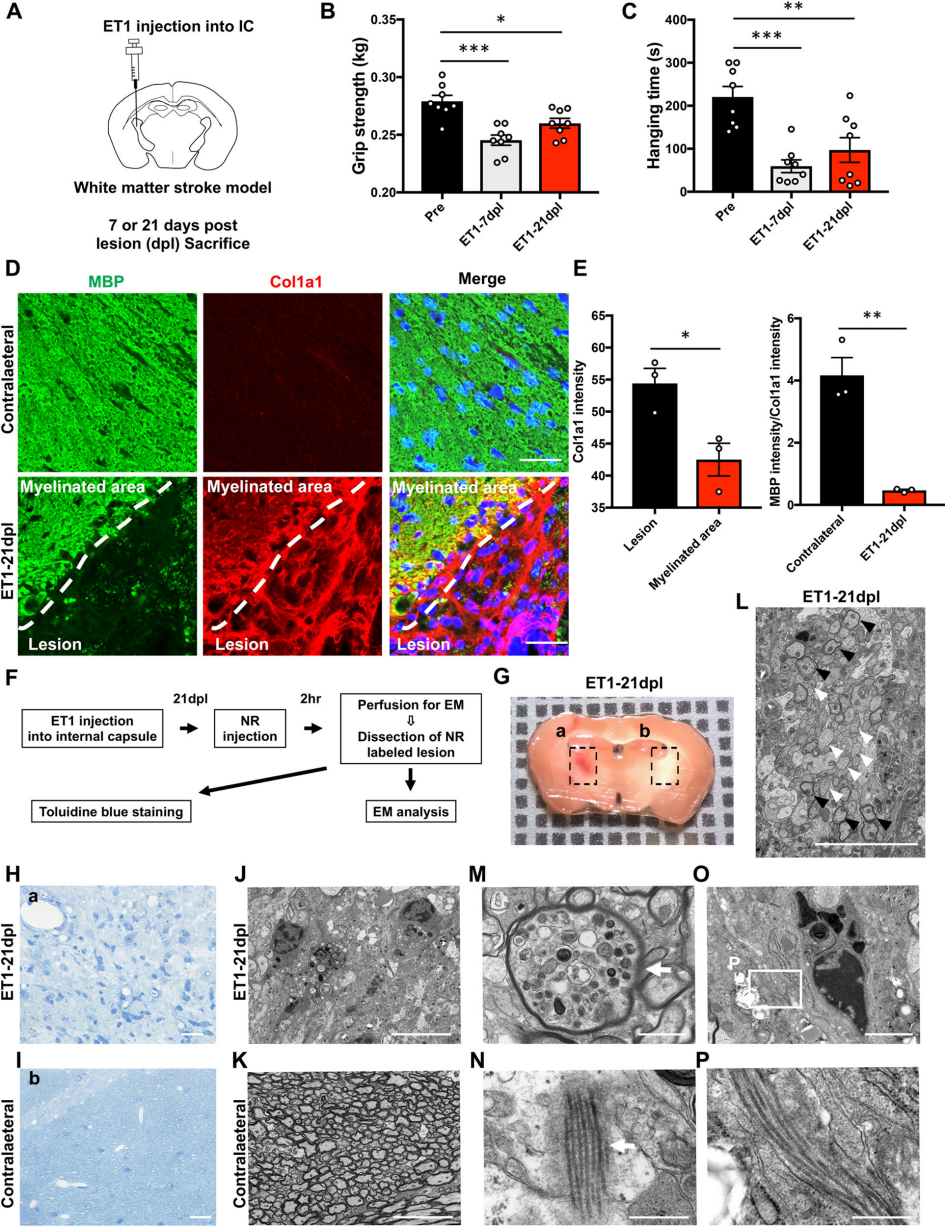
Collagen fibres accumulate in demyelinated lesions after white matter injury. **A** Schematic diagram of the white matter injury model. ET1 was injected into the internal capsule (IC). **B**, **C** Quantification of the average grip strength (**B**) and hanging time (**C**) before lesions (Pre), 7 days post-lesion (dpl), and 21 dpl after ET1 injection (*n* = 8). **D** Double immunofluorescence stained images of myelin basic protein (MBP, green) and Col1a1 (red) in coronal sections from ET1-injected mice at 21 dpl. The white dotted line indicates the border between a myelinated area and a lesion. Nuclei are counterstained with Hoechst (blue). Scale bars, 50 µm. **E** Quantification of the fluorescence intensity of Col1a1 between the lesion and myelinated area 21 dpl after ET1 injection (*n* = 3, left panel). Quantification of the fluorescence intensity ratio of MBP to Col1a1 (*n* = 3, right panel). **F** Experimental design of EM analyses. Neutral red (NR) injection 2 h before sacrifice at 21 dpl. NR-labelled lesions and contralateral IC were dissected and embedded in epoxy resin. **G** NR labelling was detected in the ipsilateral IC at 21 dpl (a; black dotted box), but not in the contralateral IC (b; black dotted box). **H**, **I** Representative images of semithin sections obtained from ET1-induced lesions (Ga; black dotted box) and the contralateral IC (Gb; black dotted box) stained with toluidine blue. Scale bars, 20 µm. **J**, **K** In the EM analysis, white matter injury is observed in an ET1-injected area at 21 dpl (**J**), and the contralateral IC has thick compact myelin (**K**). Scale bars, 10 µm. **L** Myelinated axons (black arrowheads) and demyelinated axons (white arrowheads) are present around the lesion at 21 dpl. Scale bar, 20 µm. **M**, **N** Axonal injury (white arrow in (**M**)) and secreted collagen fibres (white arrow in (**N**)) are observed in the white matter lesion. Scale bars, 1 µm. **O** Collagen fibres are present around a microglia/macrophage-like cell. Scale bar, 2 µm. **P** An enlarged image of the boxed area in (**O**). Scale bar, 500 nm. The mean ± SEM is shown as bars and lines. ^*^P < 0.05, ^**^P < 0.01, ^***^P < 0.001 by Student’s *t*-test (**E**) or one-way ANOVA followed by the Tukey-Kramer test (**B**, **C**).

Next, we examined whether chronic remyelination failure was spatially correlated with collagen deposition in a white matter injury model induced by ET1 injection into the IC of mice using stereotaxic surgery (Fig. 2A). Acute motor deficits and lasting pure motor dysfunction were confirmed by the wire hanging test and grip strength test at 7 and 21 days post-lesion (dpl) (Fig. 2B, C). Given that the pro-α1 chain of type I collagen, a triple helix consisting of two α1 chains and one α2 chain, is encoded by Col1a1 [30, 31], we conducted immunostaining using anti-MBP and anti-Col1a1 antibodies at 21 dpl to examine whether regeneration was impaired in collagen-accumulated areas (Fig. 2D). Col1a1-positive signals were not observed in the contralateral non-lesioned IC at 7 dpl. All contralateral data were obtained at 7 dpl. In contrast, Col1a1-positive signals were detected in white matter lesions. We compared the Col1a1 intensity in lesions and myelinated areas around lesions 21 dpl after ET1 injection (Fig. 2E). Interestingly, Col1a1-positive signals were decreased in myelinated areas around ischaemic lesions (Fig. 2E, left panel). Furthermore, quantitative analysis demonstrated that Col1a1 accumulated in white matter lesions after ET1 injection compared with the contralateral IC (Fig. 2E, right panel). Next, we examined whether collagen had accumulated in the chronic phase of white matter injury. Neutral red (NR) labelling, a method to visualise white matter lesions for electron microscopy (EM) (Fig. 2F) [32, 33], was observed in the ipsilateral IC at 21 dpl (Fig. 2Ga), whereas no NR labelling was observed in the contralateral IC (Fig. 2Gb). Light microscopic observation of semi-thin sections stained with toluidine blue and EM observation revealed that the NR-labelled ipsilateral IC had white matter damage caused by focal ischaemia, which was not observed in the contralateral IC (Fig. 2H-K). In NR-labelled IC, demyelinated axons among myelinated axons, and axonal degeneration characterised by axonal swelling along with the accumulation of axonal organelles were observed around the lesion (Fig. 2L, M). Furthermore, secreted collagen fibres were observed in the brain parenchyma (Fig. 2N) and around microglia/macrophage-like cells (Fig. 2O and P). These results suggest that collagen fibres were secreted in the chronic lesions of white matter injury with remyelination failure.

### Collagen fibres produced by microglia/macrophages in white matter lesions are degraded by phagocytosis

**Fig. 3:**
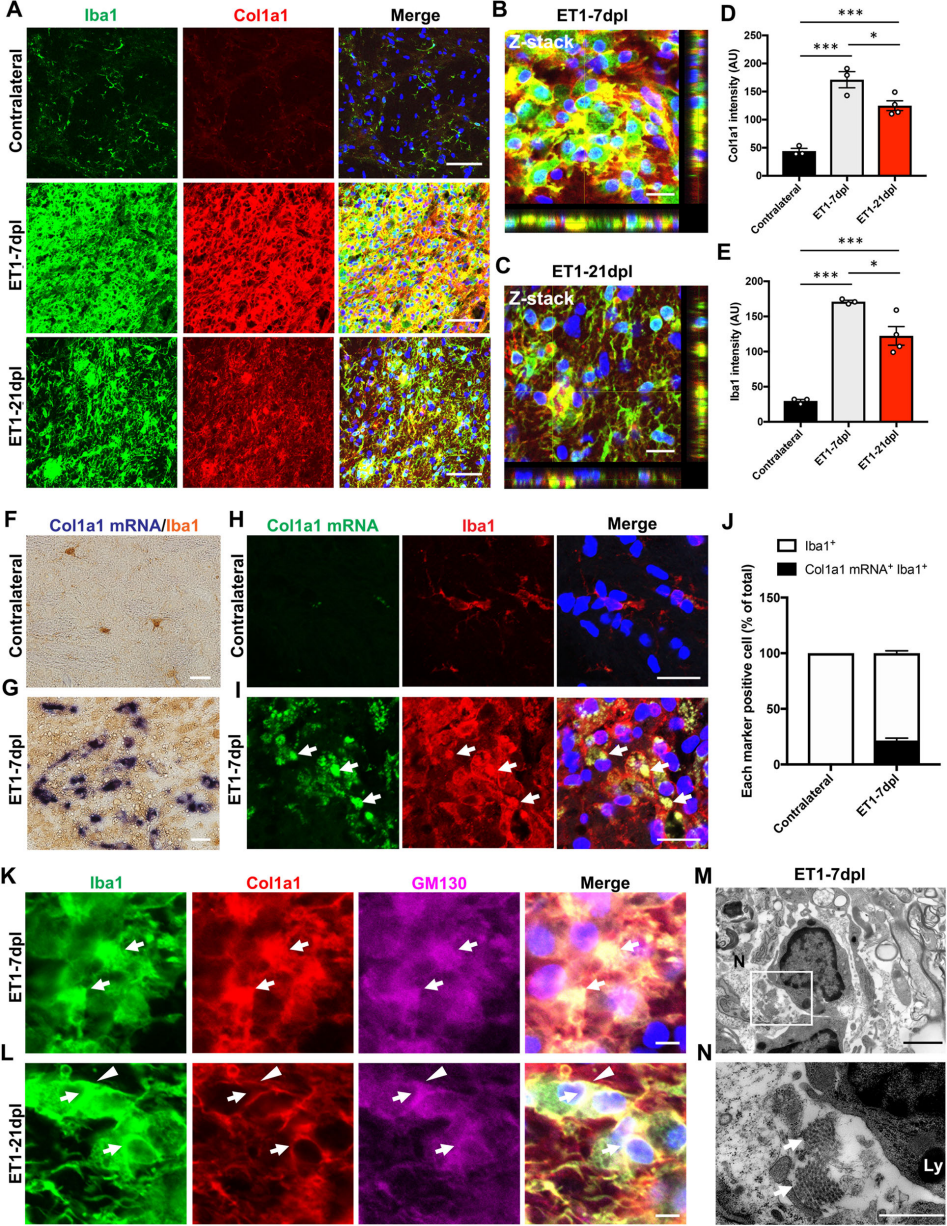
Col1a1 colocalises with Iba1-positive microglia/macrophages in ET1-induced lesions. Double immunofluorescence images of Iba1 (green) and Col1a1 (red) (**A**) and their merged Z-stack images (**B**, **C**) in internal capsule (IC) lesions after ET1 injection and the contralateral IC at 7 dpl (**A**), and ET1-lesioned IC at 7 (**A**, **B**) and 21 dpl (**C**). Scale bars, 50 (**A**) or 20 µm (**B**, **C**). Quantification of fluorescence intensity of Col1a1 (**D**) and Iba1 (**E**) staining in the contralateral IC at 7 dpl and ET1-lesioned IC at 7 and 21 dpl (Contralateral, ET1-7 dpl *n* = 3; ET1-21 dpl *n* = 4). **F**, **G** Immunohistochemistry of Iba1 after in situ hybridization (ISH) for *Col1a1* mRNA in the contralateral IC or ET1-lesioned IC at 7 dpl. ISH signals of *Col1a1* mRNA (blue) are colocalised with Iba1 (brown) at 7 dpl. Scale bars, 20 µm. **H**, **I** Immunofluorescence staining for Iba1 after fluorescent ISH for *Col1a1* mRNA in the contralateral IC or ET1-lesioned IC at 7 dpl shows that ISH signals of *Col1a1* mRNA (green) are colocalised with Iba1 immunoreactivity (red) (I, white arrows). Scale bars, 20 µm. **J** Percentage of cells double-positive for *Col1a1* mRNA and Iba1 immunoreactivity in total Iba1-positive cells (contralateral, *Col1a1* mRNA^+^ Iba1^+^; 0.00%; ET1-7 dpl, *Col1a1* mRNA^+^ Iba1^+^; 21.50 ± 2.16%, *n* = 3 mice). **K**, **L** Triple immunofluorescence staining for Iba1 (green), Col1a1 (red), and GM130 (magenta) in the ET1-lesioned IC at 7 and 21 dpl. Col1a1 immunoreactivity was colocalised with GM130-positive Golgi apparatus in Iba1-positive cells at 7 dpl (**K**, white arrows). Col1a1 signals are present in the cytoplasm (**L**, white arrows) and cell membrane (**L**, white arrowheads) of Iba1-positive cells at 21 dpl. Scale bars, 10 µm. **M** EM demonstrates a monocyte-like cell at 7 dpl. Scale bar, 2 µm. **N** An enlarged image of the boxed area in (**M**). Collagen fibres are present around a monocyte-like cell (white arrows). Ly Lysosome. Scale bar, 1 µm. Nuclei are counterstained with Hoechst (blue). ^*^P < 0.05, ^**^P < 0.01, ^***^P < 0.001 by one-way ANOVA followed by the Tukey-Kramer test (**D**, **E**).

We demonstrated that Col1a1-positive signals colocalised with Iba1-positive cells in the lesions of stroke patients (Fig. 1). We therefore hypothesised that activated microglia/macrophages produce type I collagen in white matter lesions. Double immunofluorescence staining for Iba1, a microglia/macrophage marker, and Col1a1 showed that Iba1 and Col1a1 signals were colocalised in the IC lesions at 7 and 21 dpl, which was confirmed by 3D analysis using Z-stack images (Fig. 3A-C). Quantitative analysis demonstrated the fluorescence intensity levels of Col1a1 and Iba1 signals in the ipsilateral IC at 7 dpl were significantly higher than those in the contralateral IC (Fig. 3D, E). Although the intensity of Col1a1 and Iba1 in the ipsilateral IC at 21 dpl was lower than that at 7 dpl, Iba1 and Col1a1 signals remained high in lesions at 21 dpl. To investigate whether Iba1-positive microglia/macrophages in lesions contained *Col1a1* mRNA, we performed two types of in situ hybridization (ISH), because histochemical analysis is better for cell counting and fluorescence ISH is useful for detecting colocalization. *Col1a1* mRNA was detected in Iba1-positive microglia/macrophages in the ipsilateral IC at 7 dpl but not in the contralateral IC (Fig. 3F-I). *Col1a1* mRNA signals were not detected in the negative controls (Fig. S2A, B). Furthermore, *Col1a1* mRNA-positive microglia/macrophages were present in the lesion, and approximately 20% of Iba1-positive cells were *Col1a1*-positive cells (Fig. 3J). We also found TMEM119-positive microglia at 7 dpl (Fig. S2C). Procollagen-I, a precursor of collagen and large secreted protein, accumulates in the endoplasmic reticulum, passes through the Golgi apparatus, undergoes various modifications, and is secreted from cells [31, 34]. We performed triple immunostaining for Iba1, Col1a1, and GM130, a marker of the Golgi apparatus. GM130 signals in Iba1-positive cells colocalised with Col1a1 signals in lesions at 7 and 21 dpl (Fig. 3K, L). Furthermore, collagen fibres were observed around monocyte-like cells by EM (Fig. 3M). Cerebral infarction causes cerebral oedema after vasoconstriction, leading to the excess accumulation of fluid in the intracellular or extracellular spaces of the brain [4, 35]. Collagen fibres were present in intracellular and extracellular spaces after the induction of oedema (Fig. 3N). Therefore, these results suggest that collagen fibres were secreted by microglia/macrophages in the oedematous extracellular spaces.

**Fig. 4:**
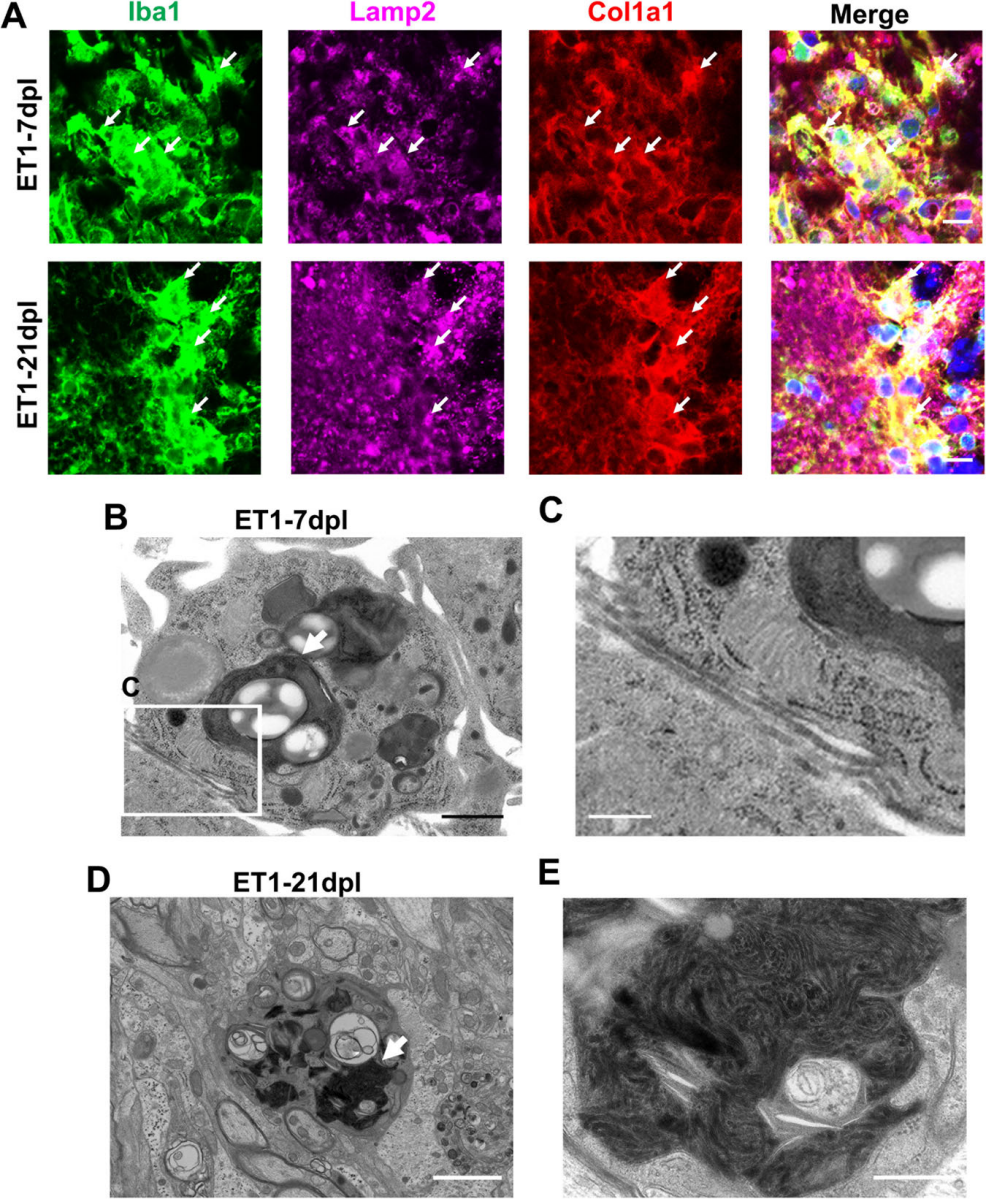
Collagen fibres are incorporated into lysosomes. **A** Triple immunofluorescence images of the ET1-lesioned IC at 7 and 21 dpl labelled with anti-Iba1 (green), anti-Col1a1 (red), and anti-LAMP2 (magenta) antibodies. Col1a1 immunoreactivity was colocalised with lamp2-positive lysosomes in Iba1-positive cells at 7 dpl (white arrows). Scale bars, 10 µm. **B**–**E** EM images of the ET1-lesioned IC at 7 and 21 dpl. Lysosomes are indicated by white arrows at 7 and 21 dpl. **C** Enlarged image of the boxed area in (**B**). Collagen fibres are present around microglia/macrophage-like cells at 7 dpl. **E** Enlarged image of the lysosome in (**D**). Collagen fibre-like structures are present in the lysosome at 21 dpl. Scale bars, 1 µm (**B**), 500 nm (**C**, **E**), or 2 µm (**D**).

Type I collagen is degraded by phagocytosis or enzymatic reactions via matrix metalloproteinase 1 [30, 31] and microglia/macrophages with high phagocytic capacity are involved in white matter repair [36–38]. Therefore, we hypothesised that microglia/macrophages reduce collagen in lesions by phagocytosis. We performed triple immunostaining using antibodies to Iba1, Col1a1, and LAMP2, a lysosomal marker (Fig. 4A). Col1a1-positive signals colocalised with LAMP2-positive signals in microglia/macrophages 7 and 21 dpl after ET1-induced white matter damage. EM was performed to observe decomposed collagen in the lysosomes of microglia/macrophages (Fig. 4B-E). Collagen fibres were present around lysosome-rich microglia/macrophage-like cells at 7 dpl. A previous study reported collagen fibre-like structures were present in cells after the phagocytosis of collagen [39, 40]. We found abnormal collagen fibre-like structures were present in the lysosomes of microglia/macrophage-like cells at 21 dpl (Fig. 4D, E). Therefore, collagen fibres may be incorporated into lysosomes for degradation. These results indicated that collagen fibres in white matter lesions were reduced by phagocytosis.

### MDM produce type I collagen in white matter lesions

**Fig. 5:**
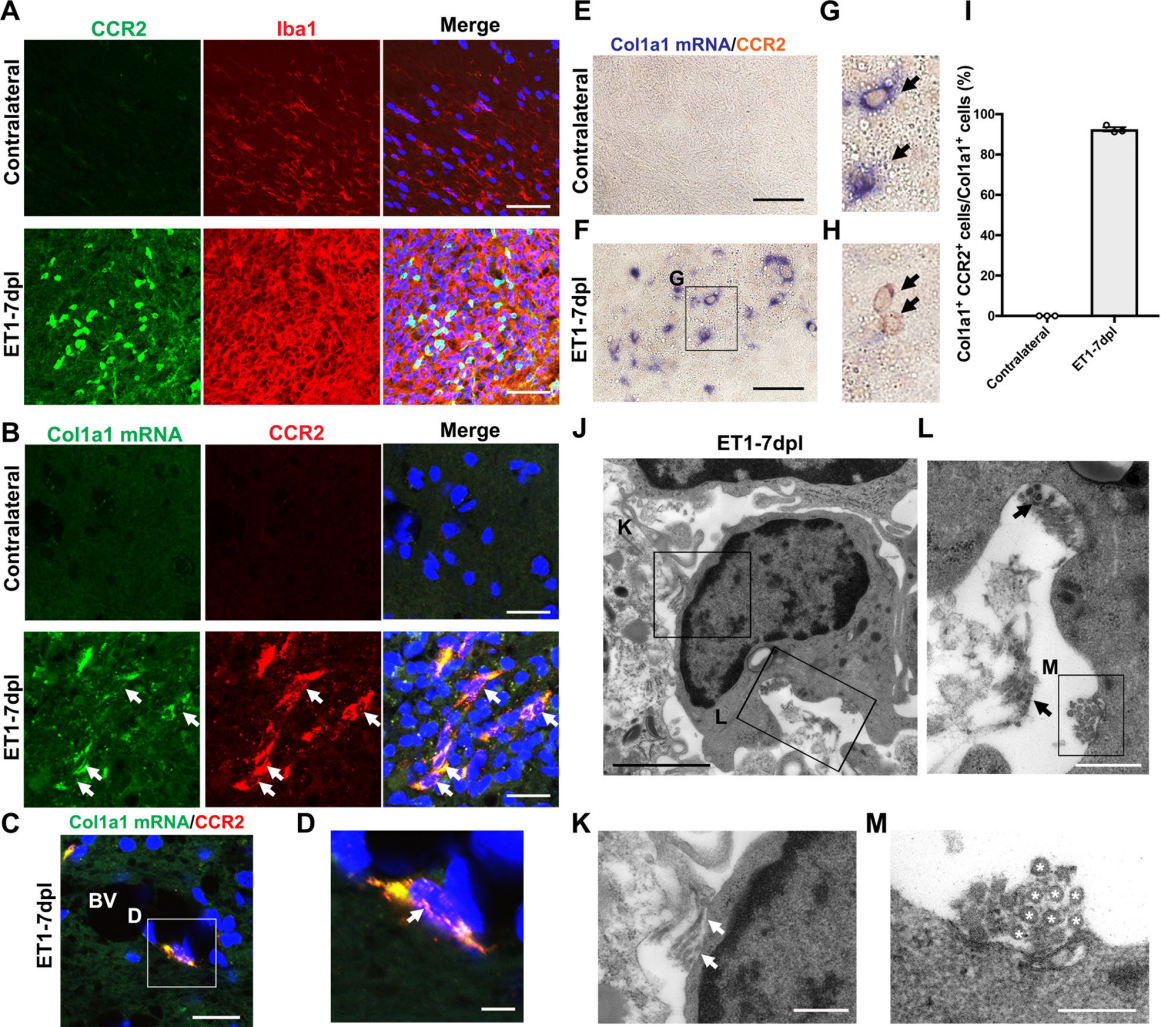
CCR2-positive macrophages produce type I collagen in white matter lesions. **A** Double immunofluorescence images of the contralateral internal capsule (IC) and ET1-lesioned IC at 7 dpl labelled with anti-CCR2 (green) and anti-Iba1 (red) antibodies. Scale bars, 50 µm. **B** Immunofluorescence staining after fluorescent in situ hybridization (ISH) for *Col1a1* mRNA in the contralateral IC or ET1-lesioned IC at 7 dpl shows that ISH signals of *Col1a1* mRNA (green) are colocalised with CCR2 immunoreactivity (red) (white arrows). Scale bars, 20 µm. **C**, **D** A *Col1a1* mRNA^+^ and CCR2^+^ double positive cell is observed around a blood vessel (BV) (d, white arrow). **D** The enlarged image of the boxed area in (**C**). Scale bars, 20 (**C**) or 10 µm (**D**). Nuclei are counterstained with Hoechst (blue). **E**, **F** Immunohistochemistry after ISH for *Col1a1* mRNA in the contralateral IC or ET1-lesioned IC at 7 dpl shows that ISH signals of *Col1a1* mRNA (blue) are colocalised with CCR2 (brown). Scale bars, 50 µm. **G** Enlarged image of the boxed area in (**F**). *Col1a1* mRNA and CCR2 double-positive cells are observed in the lesion (black arrows). **H** CCR2-positive and *Col1a1* mRNA-negative cells are present around the lesion (black arrows). **I** The percentage of *Col1a1* mRNA and CCR2 double-positive cells per *Col1a1* mRNA positive cell was calculated from three mice. **J**–**M** EM analysis indicating a monocyte-like cell appears to produce collagen fibres at 7 dpl (white arrows in (**K**), black arrows in (**L**)) in enlarged images of the boxed areas in (**J**). **M** An enlarged image of the boxed area in (**L**) shows a bundle of collagen fibres (asterisks). Scale bars, 2 µm (**J**), 500 nm (**K**, **L**), or 200 nm (**M**).

Previously, we reported that perivascular macrophages produced type I collagen around cerebral small vessels in a spontaneous hypertensive rat model [41]. We then focused on the type of macrophage present in ischaemic lesions. MDM were reported to infiltrate into lesions from peripheral blood in ischaemic stroke and atherosclerosis, and C-C chemokine receptor (CCR2) is often used as a major marker of MDM [42–44]. Therefore, we hypothesised that MDM produce collagen fibres. We performed immunofluorescence staining using an anti-CCR2 antibody as an MDM marker to distinguish it from microglia [45–47]. CCR2- and Iba1-double positive MDM were present in lesions (Fig. 5A). To examine whether CCR2-positive MDM in lesions contained *Col1a1* mRNA, we performed ISH for *Col1a1* mRNA combined with CCR2 immunostaining. *Col1a1* mRNA signals colocalised with CCR2-positive MDM in the ipsilateral IC at 7 dpl, but not in the contralateral IC (Fig. 5B-H). *Col1a1* mRNA-positive MDM were also present around blood vessels (Fig. 5C, D). ISH analysis demonstrated some *Col1a1* mRNA-negative MDM were immunostained for CCR2 in the lesion (Fig. 5H). Quantitative analysis indicated the percentage of *Col1a1*-positive cells in total CCR2-positive cells was 92.5% (Fig. 5I). Furthermore, at 7 dpl, we observed a monocyte-like cell that produced collagen fibres (Fig. 5J, K), which were present around monocyte-like cells (Fig. 5L, M). These results demonstrated that collagen fibres were secreted by MDM in white matter lesions.

Next, we performed immunostaining for CD206, an anti-inflammatory macrophage marker. CD206 signals colocalised with those of Col1a1 in white matter lesions at 7 dpl (Fig. S3A, B). *Col1a1* mRNA signals colocalised with CD206-positive macrophages at 7 dpl, but not in the contralateral IC (Fig. S3C-E). EM demonstrated perivascular macrophages (PVM), collagen fibres, and lysosomes were present around perivascular spaces (Fig. S3F-H). These results suggest that PVM produce collagen fibres around blood vessels, and that CD206-positive macrophages might secrete collagen fibres in white matter lesions. In addition, we performed immunofluorescence staining using anti-Col1a1 and anti-S100β antibodies as an astrocyte marker. However, most Col1a1-positive signals were not colocalised with anti-S100β signals (Fig. S4A-C). Interestingly, S100β-positive signals were strongly detected outside the lesions, and reactive astrocytes accumulated in perilesional areas 7 dpl after ET1 injection (Fig. S4D). In contrast, astrocytes were observed in the lesion at 21 dpl (Fig. S4A, C). *Col1a1* mRNA was not detected in astrocytes at 7 dpl (Fig. S4E). These results suggest reactive astrocytes infiltrate into white matter lesions from 7 to 21 dpl without type I collagen production. It was previously reported that perivascular cells produce some types of collagen during brain ischaemia to protect blood vessels [48, 49]. We found that Col1a1-positive signals colocalised with desmin-positive pericytes and vascular smooth muscle cells in ischaemic lesions at 7 dpl (Fig. S4F, G). Furthermore, EM demonstrated collagen fibres were present around blood vessels at 7 and 21 dpl (Fig. S4H-J). These results indicated perivascular cells secrete collagen in white matter lesions.

### Type I collagen prevents functional recovery and remyelination after IC demyelination

**Fig. 6:**
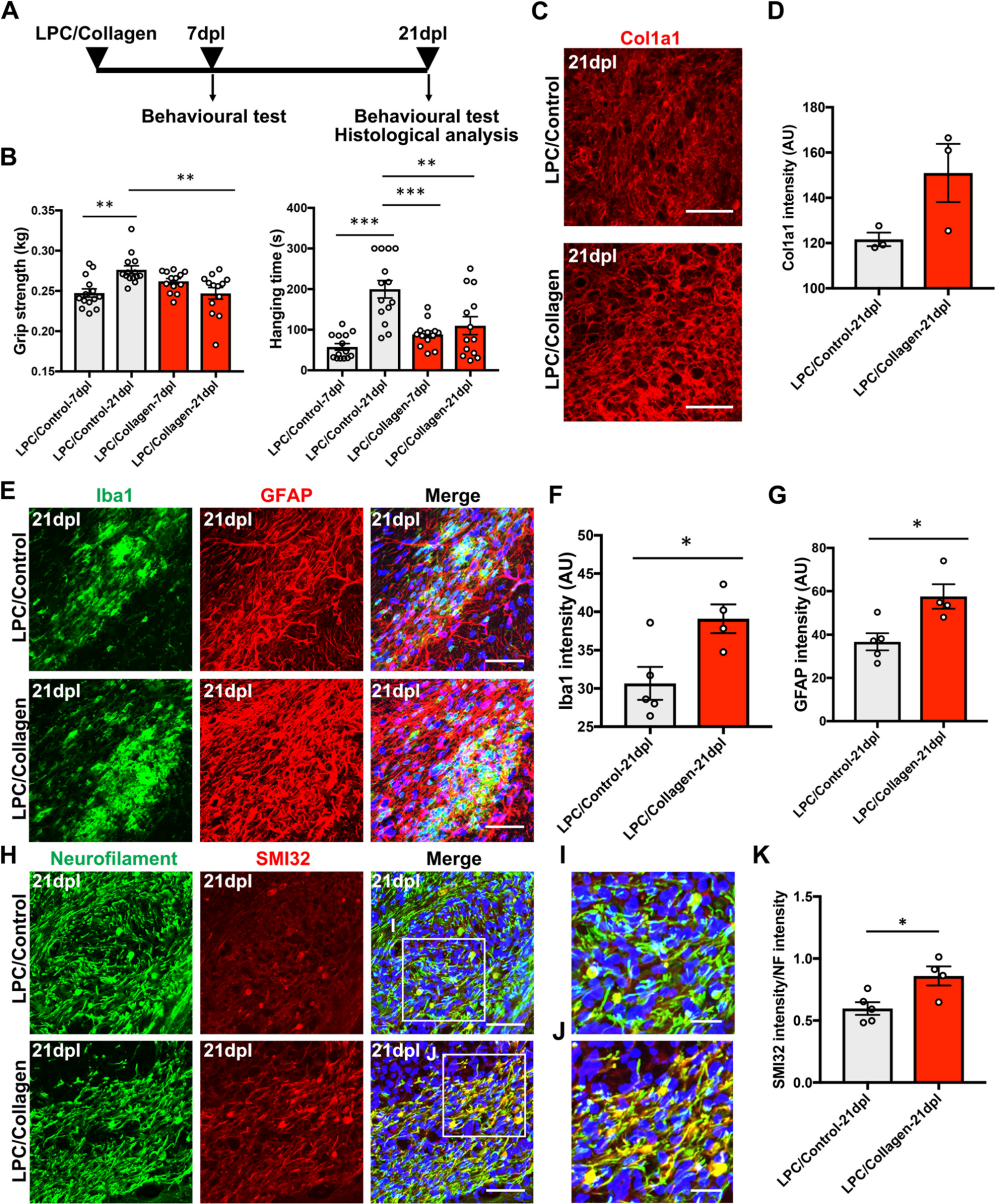
Type I collagen prevents motor recovery and exacerbates neuroinflammation after lysolecithin (LPC)-induced demyelination. **A** Experimental design for the injection of lysolecithin (LPC) with (LPC/Collagen) or without (LPC/Control) type I collagen. After injection of LPC/Collagen or LPC/Control into the internal capsule (IC), behavioural tests were performed at 7 and 21 dpl to assess motor recovery. **B** Comparison of the average grip strength and hanging time between the LPC/Collagen and LPC/Control groups (LPC/Control, *n* = 14; LPC/Collagen, *n* = 13). **C** Immunofluorescence staining for Col1a1 (red) in the IC of LPC/Control and LPC/Collagen groups at 21 dpl. Scale bars, 50 µm. **D** Quantification of fluorescence intensity based on Col1a1 staining in the IC of LPC/Control and LPC/Collagen groups at 21 dpl (LPC/Control, *n* = 3; LPC/Collagen, *n* = 3). **E** Double immunofluorescence staining for Iba1 (green) and GFAP (red) in the IC of LPC/Control and LPC/Collagen groups at 21 dpl. Scale bars, 50 µm. **F**, **G** The fluorescence intensity of Iba1 and GFAP staining in the IC of LPC/Control and LPC/Collagen groups at 21 dpl (LPC/Control, *n* = 5; LPC/Collagen, *n* = 4). **H** Double immunofluorescence staining for total (green) and non-phosphorylated (SMI32, red) neurofilaments in the IC of LPC/Control and LPC/Collagen groups at 21 dpl. Scale bars, 50 µm. **I**, **J** Enlarged images of the boxed areas in (**H**). Scale bars, 20 µm. **K** The fluorescence intensity ratio of SMI32 to neurofilament (LPC/Control, *n* = 5; LPC/Collagen, *n* = 4). The mean ± SEM is shown as bars and lines. ^*^P < 0.05, ^**^P < 0.01, ^***^P < 0.001 by Student’s *t*-test (**F**, **G**, **K**) or one-way ANOVA followed by the Tukey-Kramer test (**B**). Nuclei are counterstained with Hoechst (blue).

To determine whether type I collagen inhibited remyelination after LPC injection, we injected a solution of LPC mixed with type I collagen (LPC/Collagen) or vehicle (LPC/Control) into the IC (Fig. 6A). First, we tested the effect of the presence of type I collagen on motor recovery after IC demyelination. The motor function of the control group was significantly increased from 7 to 21 dpl in the wire hanging test and grip strength test. In contrast, the motor function of the collagen-treated group did not recover from 7 to 21 dpl (Fig. 6B). Furthermore, the intensity of Col1a1 staining was increased (Fig. 6C, D). We also injected a solution of LPC mixed with collagenase type I-treated type I collagen into the IC to evaluate whether injection of degraded collagen led to functional recovery. The motor function of the collagenase type I-treated type I collagen group recovered significantly from 7 to 21 dpl (Fig. S5). These results suggest that type I collagen prevents functional recovery after IC demyelination.

We hypothesised that glial scar formation is exacerbated in the presence of type I collagen in white matter lesions. To examine the activation level of microglia and astrocytes, we performed immunofluorescence staining using anti-Iba1 and anti-GFAP antibodies to label microglia and astrocytes, respectively (Fig. 6E). In the LPC/Collagen injected lesions, Iba1-positive intensity and glial fibrillary acidic protein (GFAP)-positive intensity were significantly increased (Fig. 6F, G). Next, to compare the axonal injury in these lesions, we examined demyelinated axons by immunofluorescence staining using SMI32 and NF antibodies (Fig. 6H). Axonal swelling was observed in lesions of the LPC/Control and LPC/Collagen groups (Fig. 6I, J). SMI32/NF-double positive signals were significantly increased in LPC/Collagen-treated lesions (Fig. 6K). These results suggest that microglial activation and astrogliosis are significantly increased in the presence of type I collagen after IC demyelination, and that this inflammation may exacerbate axonal dystrophy.

**Fig. 7:**
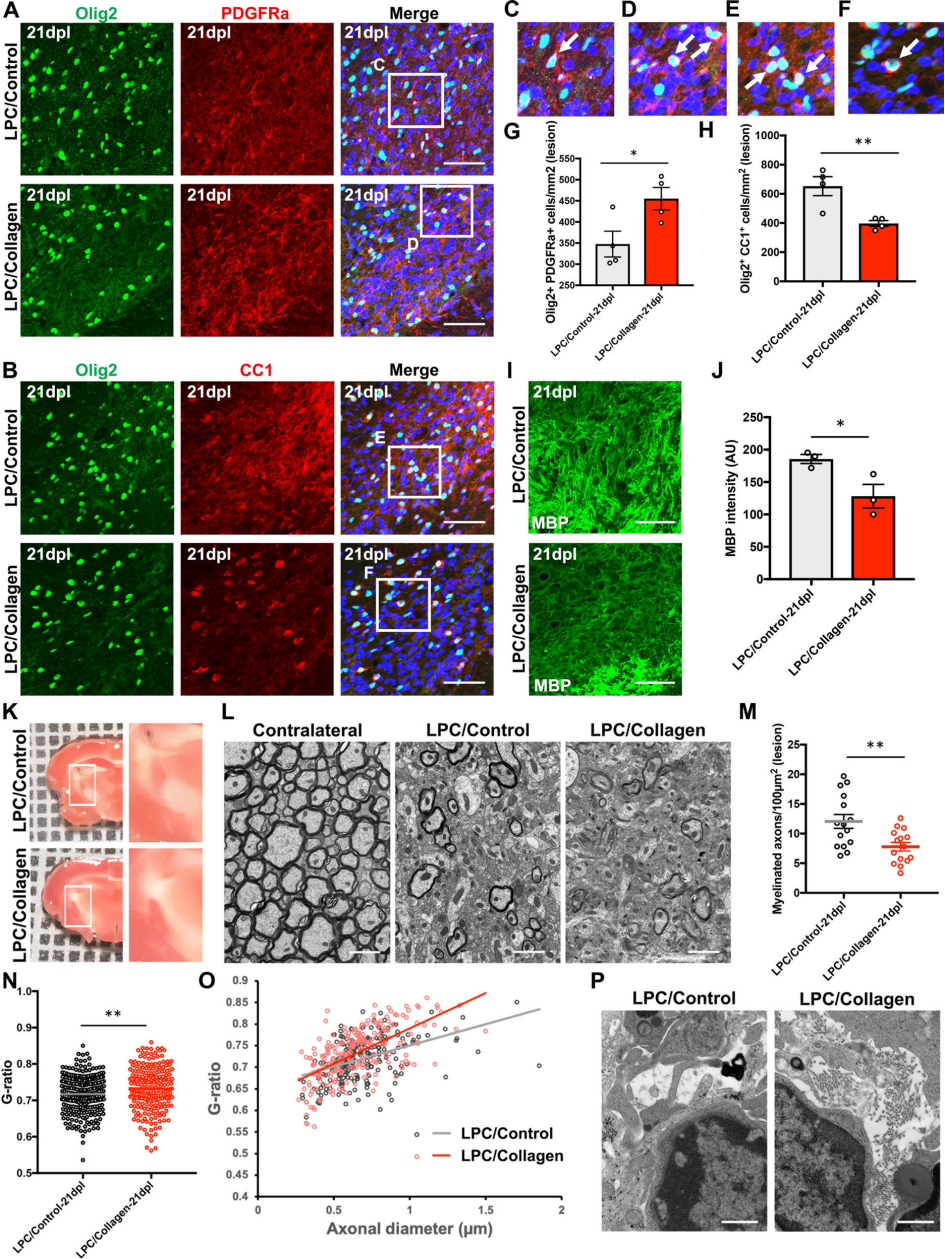
Type I collagen prevents oligodendrocyte differentiation and inhibits remyelination after lysolecithin (LPC)-induced demyelination. **A**, **B** Double immunofluorescence staining for Olig2 (green) and PDGFRα or CC1 (red) in the internal capsule (IC) after a lysolecithin (LPC) injection with (LPC/Collagen) or without (LPC/Control) type I collagen at 21 dpl. Scale bars, 50 µm. **C**–**F** Enlarged images of the boxed areas in (**A**) and (**B**). PDGFRα-positive OPC (**C**, **D**) and CC1-positive mature oligodendrocytes (**E**, **F**) are observed in lesions (arrows). **G**, **H** The density of cells positive for Olig2 and PDGFRα or Olig2 and CC1 in LPC/Control and LPC/Collagen groups at 21 dpl (LPC/Control, *n* = 4; LPC/Collagen, *n* = 4). **I** Immunofluorescence staining for myelin basic protein (MBP) (green) in the IC of LPC/Control and LPC/Collagen groups at 21 dpl. Scale bars, 50 µm. **J** Quantification of fluorescence intensity based on MBP staining in the IC of LPC/Control and LPC/Collagen groups at 21 dpl (LPC/Control, *n* = 3; LPC/Collagen, *n* = 3). **K** Neutral red labelling was observed in the IC of LPC/Control and LPC/Collagen groups at 21 dpl. **L** Representative electron microscopic images of the contralateral IC of the LPC/Control group and ipsilateral IC of the LPC/Control and LPC/Collagen groups at 21 dpl. Scale bars, 2 µm. **M** The number of myelinated axons in the LPC/Control and LPC/Collagen groups at 21 dpl (axons in visual fields: LPC/Control, *n* = 15, LPC/Collagen, *n* = 15). **N** The G-ratio in LPC/Control and LPC/Collagen groups at 21 dpl (axons in visual fields: LPC/Control, *n* = 268, LPC/Collagen, *n* = 244). **O** A scatterplot of axonal diameters and G-ratios of individual axons. **P** In electron microscopic images, collagen fibres were observed in a white matter lesion of the LPC/Collagen IC but not in a lesion of the LPC/Control IC. Scale bars, 1 µm. The mean ± SEM is shown as bars and lines. ^*^P < 0.05, ^**^P < 0.01 by Student’s *t*-test (**G**, **H**, **J**, **M**, **N**).

Next, we examine the differentiation state of oligodendrocytes in white matter lesions. We performed immunofluorescence staining for Olig2 and platelet-derived growth factor receptor alpha chain (PDGFRα) to compare OPC in lesions following LPC/Control or LPC/Collagen injection at 21 dpl (Fig. 7A, C, D). The density of OPC in lesions following LPC/Collagen injection was significantly higher than that following LPC/Control injection (Fig. 7G). The number of mature oligodendrocytes, identified by immunostaining for Olig2 and CC1 (Fig. 7B, E, F), in lesions after LPC/Collagen injection was significantly lower than that following LPC/Control injection at 21 dpl (Fig. 7H). MBP immunoreactivity in remyelinated IC lesions following LPC/Collagen injection was lower than that following LPC/Control injection at 21 dpl (Fig. 7I, J). These results indicated that type I collagen prevents oligodendrocyte differentiation in demyelinated lesions.

We performed EM to investigate the remyelination state using NR labelling to examine demyelinated lesions (Fig. 7K). NR-labelled lesions were dissected and myelin structures were examined by transmission EM. LPC-injected IC displayed demyelination compared with the contralateral IC (Fig. 7L). Next, we counted the number of myelinated axons in lesions of LPC/Control- or LPC/Collagen-injected mice. Myelinated axons were significantly decreased in the presence of type I collagen in lesions at 21 dpl (Fig. 7M). Furthermore, the G-ratio was significantly increased in LPC/Collagen-injected mice compared with that in LPC/Control-injected mice (Fig. 7N, O). In LPC/Collagen-induced lesions, collagen fibres were observed in the IC, but not in the LPC/Control-induced lesion (Fig. 7P). These results suggest that type I collagen inhibits remyelination after LPC-induced IC demyelination.

## Discussion

**Fig. 8:**
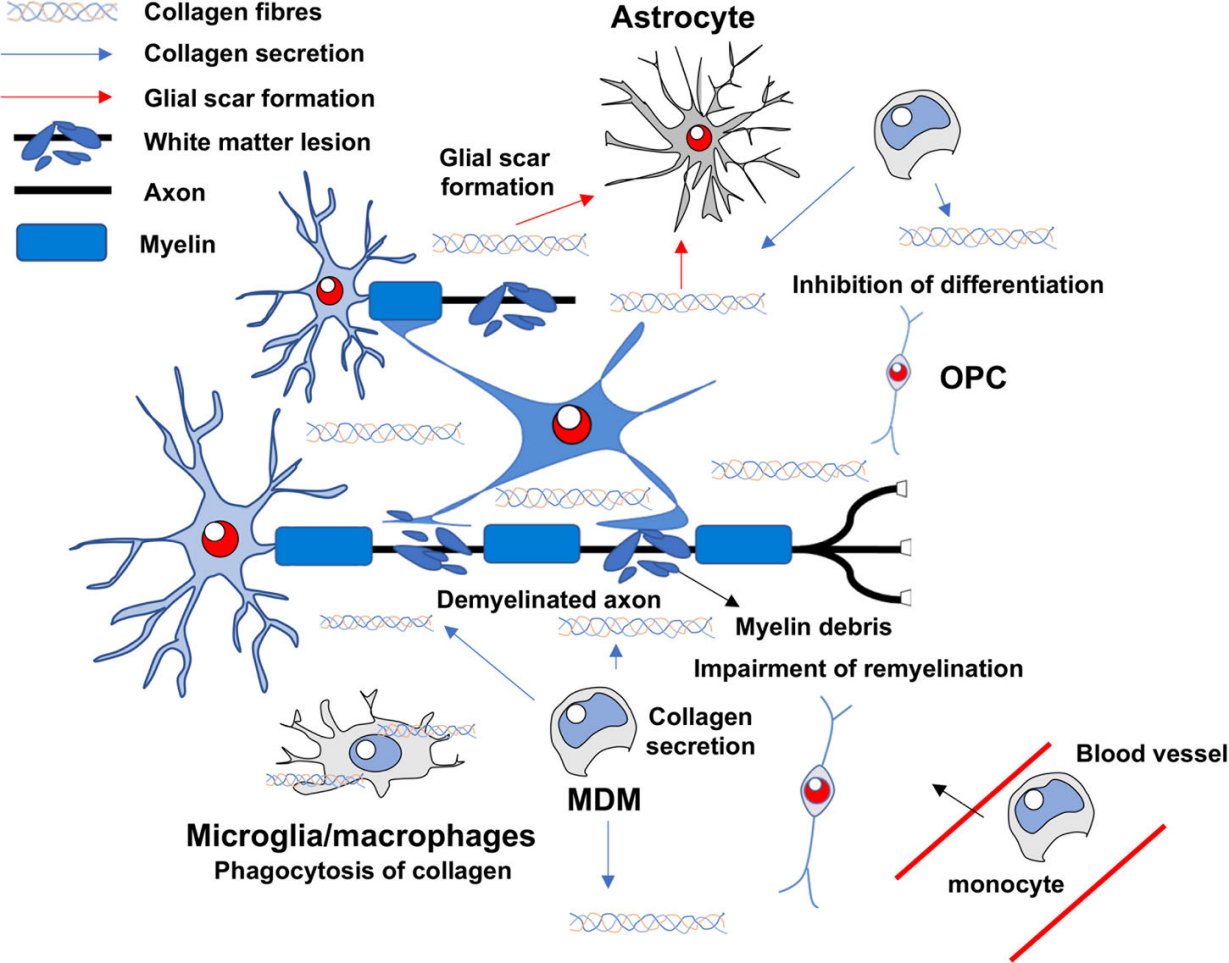
Summary of the role of Col1a1 in white matter lesions. A schematic diagram of the study findings is shown. After white matter injury, microglia/macrophages migrate into the lesions. Monocyte-derived macrophages (MDM) produce type I collagen fibres, which are phagocytosed by Iba1-positive cells. The remaining collagen inhibits the differentiation of oligodendrocyte precursor cells directly or indirectly. Collagen fibres also promote glial scar formation. Finally, white matter regeneration is impaired, and functional recovery does not occur.

In this study, we demonstrated that collagen fibres accumulated in the white matter lesions of MS patients and stroke patients. To generate a model of white matter injury, ET1, a potent vasoconstrictor, was injected into the white matter of mice. We found that type I collagen was secreted by MDM infiltrating into the CNS from the periphery. To investigate whether type I collagen inhibited white matter regeneration, LPC mixed with type I collagen was injected into the IC, which inhibited remyelination. These results suggest that type I collagen secreted in areas of white matter damage inhibited white matter regeneration (Fig. 8).

Recently, the role of the extracellular matrix in the development of the CNS and various pathological conditions has been discussed and is beginning to show promise as a new therapeutic target for neurological diseases [50, 51]. Although the post-mortem analysis of brains of MS patients indicated the upregulation of collagen-related genes in lesions, the identity of the collagen-producing cells is unknown [27, 28]. Furthermore, collagen fibres in active lesions are difficult to detect, and previous studies examined these using autopsied brains. In this study, we also demonstrated that collagen fibres accumulated at different stages of disease using biopsied lesions sampled from MS brains and postmortem brains of stroke patients. Then, we focused on the accumulation of type I collagen in white matter lesions and found that type I collagen was secreted specifically in areas where remyelination was inhibited. We showed that type I collagen was secreted by MDM in white matter lesions after blood-brain barrier breakdown.

This study used two animal models. The ET1 and LPC models are thought to produce different amounts of collagen because they cause different levels of white matter damage and ischaemia. Thus, we think that the rate of collagen degradation also differs between the ET1 and LPC models. These differences may determine whether regeneration and functional recovery occur. We also showed that PVM and perivascular cells produced collagen fibres around blood vessels (Fig. S3 and Fig. S4). Furthermore, collagen fibres increased in the perivascular area from 7 to 21 dpl after ET1 injection (Fig. S4H-J). However, most Col1a1-positive signals were detected in MDM at 7 dpl. Therefore, we assumed there were lower amounts of collagen fibres in perivascular areas compared with those produced by MDM at 7 dpl. However, it is still unclear whether microglia secrete collagen fibres. PVM and microglia are Iba1-positive CCR2-negative cells. Approximately 20% of Iba1-positive cells in lesions were *Col1a1*-positive (Fig. 3J). Although most Iba1-positive cells were CCR2-positive, other cell types positive for Iba1 and negative for CCR2 might be present; thus, microglia might secrete collagen fibres, depending on the extent and timing of the disease. Future studies should identify collagen-producing cells under various pathological conditions. It was previously reported that the deletion of MDM in a mouse model of MS, experimental autoimmune encephalomyelitis (EAE), delayed the onset of disease and reduced its severity [23]. MDM also contributed to spontaneous functional recovery after stroke [52]. In patients with MS or stroke, activated lymphocytes and monocytes are thought to exacerbate inflammation in the brain [53, 54]. Three types of monocytes exist in human blood: classical monocytes, non-classical monocytes, and intermediate monocytes [55]. Each of these has specific gene expressions and functions [56]. Interestingly, patients with MS have more classical monocytes and fewer non-classical monocytes compared to healthy individuals [57]. In addition, all three monocyte subsets were increased in patients with relapsing-remitting MS, indicating their role in disease pathology [58]. Furthermore, a subset of classical monocytes with specific gene expression was recently identified by blood immunophenotyping of patients diagnosed with MS [59]. These reports suggest that changes in the population and number of monocyte subtypes may influence MS pathology. Therefore, these monocyte subsets may be involved in collagen production in MS and stroke pathology. Furthermore, the fibrotic scar in spinal cord lesions in EAE was recently shown to derive from proliferative CNS fibroblasts [60]. Although we found that MDM secreted factors that inhibited regeneration, collagen fibres may also be protective in the early stages of white matter injury, because collagen prevented motor deficits as indicated by the grip strength test (Fig. S6, LPC/Collagen-7dpl). However, further research is needed to determine whether collagen is protective against acute white matter damage. A recent study showed that the ablation of Col1a1-positive fibroblasts located in the meninges and perivascular space in the CNS exacerbated intracerebral haemorrhage [61]. However, we demonstrated that remyelination and functional recovery were prevented in the presence of type I collagen after IC demyelination. Taken together, we concluded that white matter regeneration was finally inhibited by the secretion of type I collagen after white matter injury.

Under pathological conditions, activated macrophages can be classified into pro-inflammatory and anti-inflammatory types, which have distinct gene expression patterns [62, 63]. Anti-inflammatory macrophages are thought to promote oligodendrocyte differentiation [17, 20]. We showed that CD206-positive macrophages produced type I collagen around injured axons. Therefore, type I collagen may be secreted as an anti-inflammatory factor to protect demyelinated axons. However, the remyelination of demyelinated axons may be prevented by physical barriers related to the extracellular matrix surrounding the axons. As the balance between pro-inflammatory and anti-inflammatory macrophages has an important role in pathogenesis, this study suggests that several factors are involved in the altered lesion environment associated with the influx of MDM. It was reported that microvascular endothelial cells enhanced the autophagy-lysosome pathway by engulfing myelin debris, and these abnormalities in debris clearance induced fibrosis in the spinal cord [64]. Furthermore, the clearance of myelin debris by microglia promoted remyelination [65]. We found that myelin debris and abnormal collagen-like structures remained inside lysosomes in microglia/macrophages. Thus, the dysfunctional autophagy-lysosome pathway may also be involved in the pathogenesis of white matter injury because of the excessive phagocytosis of collagen.

Collagen forms fibrous structures by cross-linking lysyl oxidase (LOX), which is modulated by the transcription factor hypoxia-inducible factor 1 [66, 67]. Therefore, collagen fibres may be produced by LOX and *Col1a1* in ischaemic white matter lesions. We found that the injection of LPC/collagen inhibited remyelination, and collagen fibres were observed in the lesions. This might involve the synthesis of collagen fibres by an enzymatic reaction of LOX from the migrated cell population in the lesion. We also observed an exacerbation of inflammatory responses by microglia and astrocytes. Thus, collagen fibres may interact with astrocytes via the integrin-N-cadherin pathway to promote glial scar formation, resulting in sustained inflammation [68]. OPC normally migrate to demyelinated lesions early in disease and their differentiation is induced by various factors, including stimulation by microglia and astrocytes [17, 18, 20]. Therefore, it is likely that OPC differentiation was inhibited in the presence of type I collagen prior to glial scar formation. It was also previously suggested that OPC differentiation was inhibited in the presence of fibroblasts, and our results complement these reports [29].

In conclusion, our data demonstrated that type I collagen has a role in the inhibition of myelin repair following white matter damage. Therefore, collagen modulation is a novel therapeutic target for white matter injury. Research to clarify the functional mechanism of Col1a1 derived from MDM underlying the pathology of white matter injury is underway in our laboratory.

## Materials and methods

### Human brain samples

We obtained paraffin sections from postmortem brain samples of stroke patients and biopsied brain samples of MS patients from the National Center of Neurology and Psychiatry (NCNP) or Brain Research Institute at Niigata University, respectively, through Japan Brain Bank Net (JBBN). No statistical methods were used to determine the sample size. Two to three samples each of human MS and stroke tissues were used. Informed consent was obtained from all patients. Paraffin sections were examined by Klüver-Barrera staining, Azan staining, and fluorescent immunohistochemistry. All experiments for the clinical study were approved by the Ethical Committee of Jichi Medical University (approval number 23-088), NCNP (approval numbers A2021-126, XXXX-307 and A2023-114), and Niigata University (approval number C2023-0090).

### Animals

Eight-week-old male C57BL/6 J mice were purchased from Japan SLC (Hamamatsu, Japan) or Clea Japan (Fujinomiya, Japan) and maintained at the animal facility at Jichi Medical University. These mice were kept in standard cages with fewer than five mice per cage at 20 °C–25 °C on a 12 h light/dark cycle. No statistical methods were used to determine the sample size. Three to five mice were used for histological analyses. For behavioural tests, 8 to 14 mice were used. We randomly assigned mice to experimental groups without randomisation or blinding. All animal experiments were performed in accordance with the Animal Research: Reporting In Vivo Experiments guidelines. All efforts were made to use only the number of animals necessary. All experiments were approved by the Institutional Animal Care and Use Committee at Jichi Medical University (approval number 22047-01) and performed in accordance with the guidelines on the care and use of animals of this committee.

### White matter injury mouse model

White matter injury was induced by the injection of 1% LPC (Sigma-Aldrich Japan, Tokyo, Japan) in phosphate-buffered saline (PBS) or ET1 solution (Peptide Institute Japan, Osaka, Japan; 0.25 mg/ml) into the IC. Mice were injected with an anaesthetic drug mixture (0.3 mg/kg medetomidine, 4.0 mg/kg midazolam, and 5.0 mg/kg butorphanol) and placed on a stereotaxic frame (Narishige, Tokyo, Japan) using the mouse ear bar. Stereotaxis was performed according to a previously described protocol with slight modifications [25]. Briefly, LPC was stereotaxically injected into the right internal capsule (anteroposterior: −1.5 mm from the bregma; mediolateral: +2.5 mm from the midline; dorsoventral: −3.8 and −3.7 mm from the dura). A Hamilton syringe with a 33 G needle connected to a micro injector (IMS-3; Narishige) was inserted into the brain and maintained in place for 3 min before the injection of LPC, ET1, Type I collagen (R&D Systems; 200 µg/ml), or collagenase type I (Fujifilm)-treated type I collagen in LPC (total volume of 2 µl; injection of 1 µl at each depth). The interval between injections was 2 min. The needle was kept in place for 10 min after the last injection to reduce reflux along the needle track. Mice were sacrificed at 7 or 21 days post-lesion (dpl).

### Type I collagen injection

Type I collagen in 20 mM acetic acid was mixed with 1% LPC before injection into the IC. Type I collagen was degraded by collagenase type I treatment as a control for the collagen injection. Type I collagen (5 mg/ml) was incubated overnight at 37 °C in collagenase type I (5 mg/ml). Type I collagen (200 µg/ml) in 1% LPC or control solution (acetic acid in 1% LPC) or collagenase type I-treated type I collagen was injected using a stereotaxic technique. Mice were sacrificed at 21 dpl after behavioural tests were performed.

### Fluorescent immunohistochemistry

Immunofluorescence staining of cryosections was performed as described previously [69]. Mice were injected with an anaesthetic drug mixture and perfused transcardially with 2 ml/g body weight of 4% (w/v) paraformaldehyde in PBS (PFA/PBS). Brains were then rapidly dissected and immediately immersed in 4% PFA/PBS at 4 °C for overnight post-fixation. For cryoprotection, fixed brains were immersed overnight in 15% (w/v) sucrose/PBS, pH 7.4, at 4 °C, followed by overnight immersion in 30% (w/v) sucrose/PBS, pH 7.4, at 4 °C, before freezing in OCT compound (Sakura Finetek, Tokyo, Japan). Cryosections (12-µm thick) were prepared using a cryostat (CM3050; Leica Microsystems), collected on coated glass slides (Matsunami, Osaka, Japan), and stored at −30 °C. For FluoroMyelin staining, cryosections were incubated with FluoroMyelin Green (1:50; Thermo Fisher Scientific, Waltham, MA, USA) in PBS, pH 7.4, containing 0.1% Triton X-100 (TX) for 1 h at room temperature (RT). For immunofluorescence staining, cryosections were permeabilised and incubated with blocking buffer (0.3% TX/Tris-buffered saline (TBS) and 10% normal goat serum) for 1 h, and then incubated overnight at 4 °C with primary antibodies in the same buffer. Thereafter, the cryosections were incubated for 1 h with primary antibodies at RT, washed three times in 0.1% TX/TBS, and then incubated for 3 h at RT with secondary antibodies. Images were captured on a confocal microscope (FV1000; Olympus, Tokyo, Japan). For quantification, fluorescent intensities of images were examined by Fiji-imageJ.

### Behavioural tests

The grip strength test, a simple and non-invasive method for evaluating muscle force of rodents in vivo [70, 71], was performed as described previously [26]. Grip strength was measured using a grip strength metre (GMP-100; Melquest, Toyama, Japan). Mice were placed on a metal wire mesh for a few minutes and allowed to grab the mesh. Then, mice were gently pulled back, and the grip strength (force) was recorded in five trials per animal at 1 min intervals. The highest and lowest scores were removed, and the final grip strength was determined by calculating the average score of the remaining three trials.

The wire hanging test was performed to evaluate motor function and motor paralysis according to our previously described protocol with slight modifications [25]. Briefly, mice were allowed to hang from a 2 mm thick rod with both forepaws. Hanging time was counted from the start of placement on the rod to the time the mouse fell off. Two trials were conducted per mouse, and the hanging time was determined using the average score of each mouse and then averaging these for each group.

### Antibodies

Primary antibodies for immunostaining were rabbit polyclonal anti-ionised calcium binding adaptor molecule 1 (Iba1) (1:400; 019-19741, Fujifilm Wako Pure Chemical Co., Tokyo, Japan), rabbit polyclonal anti-type I collagen (1:500; LB-1102, LSL), rabbit polyclonal anti-S100β (1:1 000; ab52642, Abcam), rabbit polyclonal anti-Olig2 (1:100; 18953, IBL America, Minneapolis, USA), rabbit polyclonal anti-GM130 (1:100; SAB5700801, Sigma-Aldrich), rabbit polyclonal anti-mannose receptor (CD206) (1:1 000; ab64693, Abcam), rabbit polyclonal anti-neurofilament (NF) 200 (1:400; N4142, Sigma-Aldrich), rabbit polyclonal anti-TMEM 119 (1:100; ab209064, Abcam), rabbit monoclonal anti-CCR2 (1:100; ab216863, Abcam), rabbit polyclonal anti-desmin (1:1 000; ab15200, Abcam), rat monoclonal anti-myelin basic protein (MBP, aa-82-87) (1:100; MAB386, Merck Millipore, Billerica, MA, USA), rat monoclonal anti-Iba1 (1:400; ab283346, Abcam), rat monoclonal anti-lamp2 (1:100; ab13524, Abcam), rat monoclonal anti-platelet-derived growth factor receptor a chain (PDGFRα) (1:100; 558774, BD Biosciences, Franklin Lakes, NJ, USA), mouse monoclonal anti-Col1a1 (1:100; SAB 1402151, Sigma-Aldrich), mouse monoclonal anti-Iba1 (1:400; NCNP24, Fujifilm Wako Pure Chemical Co., Tokyo, Japan), mouse monoclonal anti-adenomatous polyposis coli (APC/CC1) (1:100; OP80, Merck Millipore), mouse monoclonal anti-glial fibrillary acidic protein (GFAP) (1:100; G3893, Sigma-Aldrich), and mouse monoclonal anti-NF H non-phosphorylated mouse mAb (SMI32) (1:1 000; 801701, BioLegend). Alexa Fluor 488- or Alexa Fluor 568- or Alexa Fluor 633-conjugated species-specific secondary antibodies (1:500; Thermo Fisher Scientific) were used, together with 1 µg/ml Hoechst 33342 (Thermo Fisher Scientific) for labelling nuclei.

### In situ hybridization (ISH) and immunohistochemistry

PCR was used to amplify DNA fragments of the *Col1a1* gene from mouse brain cDNA. The following primers were used to amplify DNA fragments of *Col1a1* (NCBI Reference Sequence: NP_031768.2): forward 5′-TGA AGT CAG CTG CAT ACA CA-3′ and reverse 5′-TGG CAC CAT CCA AAC CAC TG-3′ (716 bp). Amplified DNA fragments were ligated into the pGEM-T vector (Promega, Madison, WI, USA) and cloned. Gene-specific antisense or sense digoxigenin (DIG)-labelled cRNA probes were made using a T7 RNA polymerase (Toyobo, Osaka, Japan) and DIG RNA labelling mix (Roche Diagnostics, Penzberg, Germany).

Mouse brain sections were incubated in 10 mM citrate buffer (pH 6.0) for 15 min at 95 °C and then treated with proteinase K (0.5 μg/mL) for 5 min, and 4% PFA in 100 mM PB (pH 7.4) for 2 min. DIG-labelled cRNA probes for *Col1a1* hybridization were used at 55 °C for 16 h. *Col1a1* mRNA was visualised with alkaline-phosphatase-conjugated sheep anti-DIG or anti-fluorescein Fab fragments (1:50,000 dilution, Roche Diagnostics) by using 4-nitroblue tetrazolium chloride (NBT) and 5-bromo-4-chloro-3-indolyl phosphate (BCIP; Roche Diagnostics). Control experiments were performed, and no specific signal was detected in sections processed with the DIG-labelled sense RNA probe.

For double-staining, after *Col1a1* mRNA had been detected by ISH, sections were immunostained as described in our previous report [41]. The sections were then incubated overnight at room temperature in PBS with primary antibodies. Absence of an observable nonspecific reaction was confirmed by using normal rabbit serum. The ABC method (Vector Laboratories) was performed with 3,3’-diaminobenzidine (Dojindo Laboratories, Kumamoto, Japan) as the substrate.

### In situ hybridization chain reaction (ISHpalette™)

DNA probes against mouse *Col1a1* mRNA (Table S1) were designed with split-initiator sequences. In situ hybridization chain reaction (isHCR) was performed according to the manufacturer’s instructions (Nepa Gene, Chiba, Japan). Mouse brain sections were treated with methanol for 10 min at RT, and then prehybridization was performed using a hybridization solution containing 0.5 × SSC, 10% dextran sulphate 500,000 (Fujifilm Wako, Osaka, Japan), 0.1% Tween 20 (Promega), 50 μg/mL heparin sodium (Nacalai Tesque, Kyoto, Japan), and 1× Denhardt’s (Nacalai Tesque) for 5 min at 37 °C. After denaturing DNA probes at 95 °C for more than 5 min, all split probes for *Col1a1* were mixed and diluted with 100 nM hybridization solution. Hybridization was performed at 37 °C for 16 h. After washing the extra probes, sections were incubated with amplification buffer containing 8 × SSC, 10% dextran sulphate 500,000, 0.2% Triton X-100 (Nacalai Tesque), and 100 mM MgCl_2_ for more than 5 min. ISHpalette™ Short hairpin amplifier SaraFluor™ 488-S23 (Nepa Gene) was heated to 95 °C for 1 min and then gradually cooled to 65 °C for 15 min and to 25 °C for 40 min. Subsequently, the fluorophore-conjugated short hairpin DNA were diluted with amplification buffer (60 nM) and then applied to the sections to visualise *Col1a1* mRNA by chain reaction for 2 h. Negative control experiments for isHCR were performed without probes, and no specific signals were detected.

For double-staining, after *Col1a1* mRNA was detected by isHCR, sections were blocked with PBS containing 1% bovine serum albumin (Nacalai Tesque) for 30 min at RT and incubated in PBS with the following primary antibodies, rabbit polyclonal anti-ionised calcium binding adapter molecule 1 (Iba1, 1:400) and rabbit monoclonal anti-CCR2 (6 μg/ml), overnight at RT. For visualization, Alexa Fluor 568-conjugated goat anti-rabbit IgG (Thermo Fisher Scientific, Waltham, MA, USA) was used as a secondary antibody, and nuclei were stained with 4′,6-diamidino-2-phenylindole (0.5 μg/ml, Dojindo Laboratories). Stained sections were observed with a confocal laser microscope (FV1000, Olympus).

### Neutral red (NR) labelling of white matter lesions

Following white matter damage, 500 µl of 1% NR (10 mg/ml; Sigma-Aldrich) in PBS was injected intraperitoneally into each mouse to identify the focal lesion as described previously [32, 33]. Mice were sacrificed 2 h after the NR injection with (for electron microscopy) or without (for RNA extraction) fixation. The brains were sliced at 500 µm thickness by a vibratome (LinearSlicer Pro10, DOSAKA EM, Kyoto, Japan). Areas of NR labelling were identified under stereomicroscopes and dissected for subsequent experiments.

### Electron microscopy (EM) analysis

Briefly, 2 h after the NR injection, mice at 7 or 21 dpl were anaesthetised and transcardially perfused with PBS and then with 2% PFA/2.5% glutaraldehyde in 0.1 M phosphate buffer (PB; pH 7.4). Brain tissues were post-fixed in the same fixative at 4 °C overnight. For transmission electron microscopy (TEM), NR-labelled lesions were dissected and post-fixed in cold 2% OsO_4_ in 0.1 M PB for 90 min, dehydrated in a graded ethanol series, and embedded in Quetol 812 epoxy resin (Nisshin EM Co., Tokyo, Japan). The resin was incubated at 60 °C for two nights to ensure polymerization. Prior to TEM observation, semithin sections were cut at 1 µm thickness, stained with 1% toluidine blue, and examined under a light microscope (BX63; Olympus). Ultrathin sections were prepared with an ultramicrotome (Ultracut UCT, LEICA) and stained with uranyl acetate and lead citrate. Images were captured by TEM (HT7700; Hitachi High-Tech, Tokyo, Japan). The cell type was determined using a combination of morphology, cell size, and characterization of organelles by EM [23, 31, 36, 72–75].

### Statistical analysis

All quantitative analyses are presented as the mean ± standard error of the mean (SEM) for each mouse. For quantification, the demyelinated areas of images captured by confocal microscopy were determined by Fiji-ImageJ. No exclusion criteria were pre-determined. We quantified myelinated axons in the lesioned IC from low magnification images of three individual mice by EM (visual fields; LPC/Control, *n* = 15, LPC/Collagen, *n* = 15). The G-ratios were calculated from high magnification images of EM from three individual mice (axons in visual fields; LPC/Control, *n* = 265, LPC/Collagen, *n* = 242). Statistical analyses were performed using Prism 7 (GraphPad Software). Quantitative values are shown in the Supplementary Materials. Statistical comparisons were performed using the Student’s *t*-test or one-way ANOVA, followed by the Tukey-Kramer test. Statistical significance was indicated as follows: ^*^P < 0.05, ^**^P < 0.01, ^***^P < 0.001.

## Supplementary information

Supplementary materials

## Data Availability

All relevant data are included within this manuscript and the supplementary data. Raw data will be made available on request by qualified researchers.
