# Supplementary material for: Type I collagen secreted in white matter lesions inhibits remyelination and functional recovery
Source: Cell Death Dis. 2025 Apr 13;16(1):285. doi: 10.1038/s41419-025-07633-w (PMC11993711; doi:10.1038/s41419-025-07633-w)
Supplement: Supplementary file 1 — Supplementary materials [file 41419_2025_7633_MOESM1_ESM.pdf]

Supplementary Tables

Supplementary Table 1. Probes used for the ISH PCR of *Col1a1*

Probe set name	Sequence of sprit probe 1	Sequence of sprit probe 2
Col1a1-1S23	GGGTGGTCGaaGCAGTGGCCCCTAAGAGGAGCAGGA	ATGTCTTCTTGGCCATGCGTCAGGAaaTCGAAGTCGTAT
Col1a1-2S23	GGGTGGTCGaaATTGTGTATGCAGCTGACTTCAGGG	CGTCTCACCATTGGGGACCCTTAGGaaTCGAAGTCGTAT
Col1a1-3S23	GGGTGGTCGaaAGATCAAGCATACCTCGGGTTTCCA	ACACAGCCGTGCCATTGTGGCAGATaaTCGAAGTCGTAT
Col1a1-4S23	GGGTGGTCGaaAGCACTCGCCCTCCCGTCTTTGGGG	ATACGTATTCTTCCGGGCAGAAAGCaaTCGAAGTCGTAT
Col1a1-5S23	GGGTGGTCGaaGCAAAGTTTCCTCCAAGGCCAGGGG	TCATCATAGCCATAGGACATCTGGGaaTCGAAGTCGTAT
Col1a1-6S23	GGGTGGTCGaaAGGCACGGAAACTCCAGCTGATTTT	ACGAGGACCAGAAGGACCCATGGGGaaTCGAAGTCGTAT
Col1a1-7S23	GGGTGGTCGaaGGCAATCCACGAGCACCCTGAGGTC	TTCATTCCAGGGAGGCCAGCTGTTCaaTCGAAGTCGTAT
Col1a1-8S23	GGGTGGTCGaaCAAACCACTGAAGCCTCGGTGTCCC	AGGACCAGCATCTCCTTTGGCACCAaaTCGAAGTCGTAT
Col1a1-9S23	GGGTGGTCGaaAGGAGCACCATTGGCACCTTTAGCG	AGGGAAGCCAGGAGCACCAGCAATAaaTCGAAGTCGTAT
Col1a1-10S23	GGGTGGTCGaaACGGGCTCCTCGTTTTCCTTCTTCT	AGGCAGTCCGGAAGGTCCAGGCTCAaaTCGAAGTCGTAT
Col1a1-11S23	GGGTGGTCGaaAACACCATCAGCACCAGGGAAACCA	TTCACCGGAAGGACCCTTGGGGCCAaaTCGAAGTCGTAT
Col1a1-12S23	GGGTGGTCGaaGAATCCCATCACACCAGCCTGGCCA	TTCTCCAGCGGTACCCTTAGGTCCAaaTCGAAGTCGTAT
Col1a1-13S23	GGGTGGTCGaaGGGACCTTGTTCACCTCTCTCACCA	AAGACCCTGGAATCCAGGGGAGCCAaaTCGAAGTCGTAT
Col1a1-14S23	GGGTGGTCGaaGGCTTGCCTGCTTCACCAGGAGGAC	AGGTCTCCAGGAACACCCTGTTCACaaTCGAAGTCGTAT
Col1a1-15S23	GGGTGGTCGaaTTCACCAGGCATTCCCTGAAGACCG	AGGACCTGGAAGACCAGCTGCACCAaaTCGAAGTCGTAT
Col1a1-16S23	GGGTGGTCGaaGACCAGCATCACCTCTGTCACCCTT	TACCAGGAGAACCATCAGCACCTTTaaTCGAAGTCGTAT
Col1a1-17S23	GGGTGGTCGaaCCAGGTTGGCCATCAGCACCAGGGG	CCAGTATCACCAGGTTCACCTTTCGaaTCGAAGTCGTAT
Col1a1-18S23	GGGTGGTCGaaAGGAGCACCAACGTTACCAATGGGG	AGCAGCACCACGAGGACCTTTGGGTaaTCGAAGTCGTAT
Col1a1-19S23	GGGTGGTCGaaAGATCCTTTCTCACCAGCAGGACCG	AGAGCCAGCAGGTCCATCAGCACCAaaTCGAAGTCGTAT
Col1a1-20S23	GGGTGGTCGaaATACCCTGAGGTCCAGGGGTACCAG	AGACCGACCACACCACGTTGTCCAGaaTCGAAGTCGTAT
Col1a1-21S23	GGGTGGTCGaaGCCATTCTTGCCAGCGGGACCAACA	ACCAGCAGGACCAGTCTCACCACGAaaTCGAAGTCGTAT
Col1a1-22S23	GGGTGGTCGaaCTGTCTCACCCTTGTCACCACGGGG	CCTTTATGCCTCTGTCACCTTGTTCaaTCGAAGTCGTAT
Col1a1-23S23	GGGTGGTCGaaGTCTTTGCCAGGAGAACCAGCAGAG	AATGGGGCCAGGGAGACCGTTGAGTaaTCGAAGTCGTAT
Col1a1-24S23	GGGTGGTCGaaGGCCACCATCTTGAGACTTCTCTTG	CGTTAGCATCATCGGCCCGGTAGTAaaTCGAAGTCGTAT
Col1a1-25S23	GGGTGGTCGaaACCTCAAGGTCACGGTCACGAACCA	TGACTCAGGCTCTTGAGGGTGGTGTaaTCGAAGTCGTAT
Col1a1-26S23	GGGTGGTCGaaTCAGAGTGGCACATCTTGAGGTCGC	TCGATCCAGTACTCTCCGCTCTTCCaaTCGAAGTCGTAT
Col1a1-27S23	GGGTGGTCGaaGTCCAGGTTGCAGCCTTGGTTAGGG	CTCCATGTTGCAGTAGACCTTGATGaaTCGAAGTCGTAT
Col1a1-28S23	GGGTGGTCGaaTAGGGAACACACAGGTCTGACCTGT	AGTTCTTCTGAGGCACAGACGGCTGaaTCGAAGTCGTAT
Col1a1-29S23	GGGTGGTCGaaTCCTTGGGGTTCGGGCTGATGTACC	CTCTCTCCAAACCAGACGTGCTTCTaaTCGAAGTCGTAT
Col1a1-30S23	GGGTGGTCGaaGTCTGCTGGTCCATGTAGGCTACGC	AGGAGCAGGGCCTTCTTGAGGTTGCaaTCGAAGTCGTAT
Col1a1-31S23	GGGTGGTCGaaGAGCTCGATCTCGTTGGATCCCTGG	GGTGAAGCGACTGTTGCCTTCGCCTaaTCGAAGTCGTAT
Col1a1-32S23	GGGTGGTCGaaTTGGTGGTTTTGTATTCGATGACTG	ACATCGATGATGGGCAGGCGGGAGGaaTCGAAGTCGTAT
Col1a1-33S23	GGGTGGTCGaaTGGGGCACCAATGTCCAAGGGAGCC	GCCAATGTCTAGTCCGAATTCCTGGaaTCGAAGTCGTAT
Col1a1-34S23	GGGTGGTCGaaGAGGGAGTTTACACGAAGCAGGCAG	TGGGTGGGAGGGAACCAGATTGGGGaaTCGAAGTCGTAT
Col1a1-35S23	GGGTGGTCGaaTTTCCAGGGTTGGGGAAAAGTGGGC	GGAAATTGAGTTTGGGTTGTTCGTCaaTCGAAGTCGTAT
Col1a1-36S23	GGGTGGTCGaaAAGCAAGAGGACCAAGCTTCCTTTT	AAGGGACTTATACCCACATAGGTCTaaTCGAAGTCGTAT
Col1a1-37S23	GGGTGGTCGaaGGGTATCATAAGCCAAGTGGGCAGG	GAGAAAGGAGCAGAAAAGGCAGCATaaTCGAAGTCGTAT

Red letters and small case (aa) in the probe sequences indicate partial initiator and spacer sequence, respectively.

Supplementary Figures

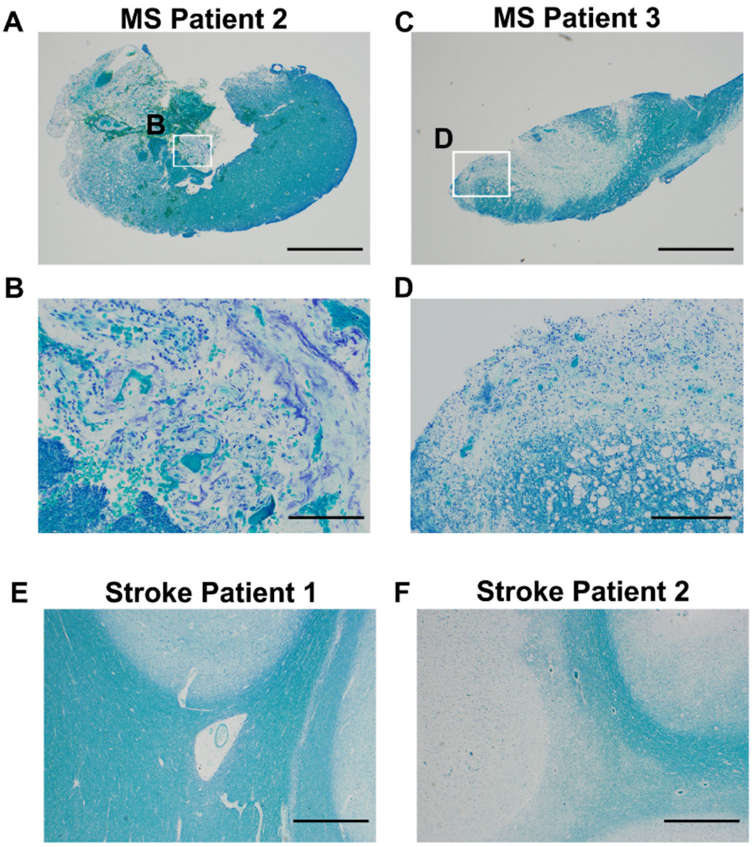

Supplementary Fig. S1 Human patients with MS or stroke show demyelination of the white matter. (A-D) Klüver-Barrera stained images of paraffin sections from MS patients. Demyelinated lesions and white matter with remaining myelin (blue stained area) are observed. (A) An active lesion from patient 2 with MS shows demyelination. Scale bar, 1 mm. (B) An enlarged image of the boxed area in (A). Scale bar, 100 μm. (C) A chronic lesion from patient 3 with MS shows demyelination. Scale bar, 1 mm. (D) An enlarged image of the boxed area in (C). Scale bar, 200 μm. (E, F) Klüver-Barrera stained images of paraffin sections from stroke patients. White matter lesions are observed in patients 1 and 2. Scale bars, 1 mm.

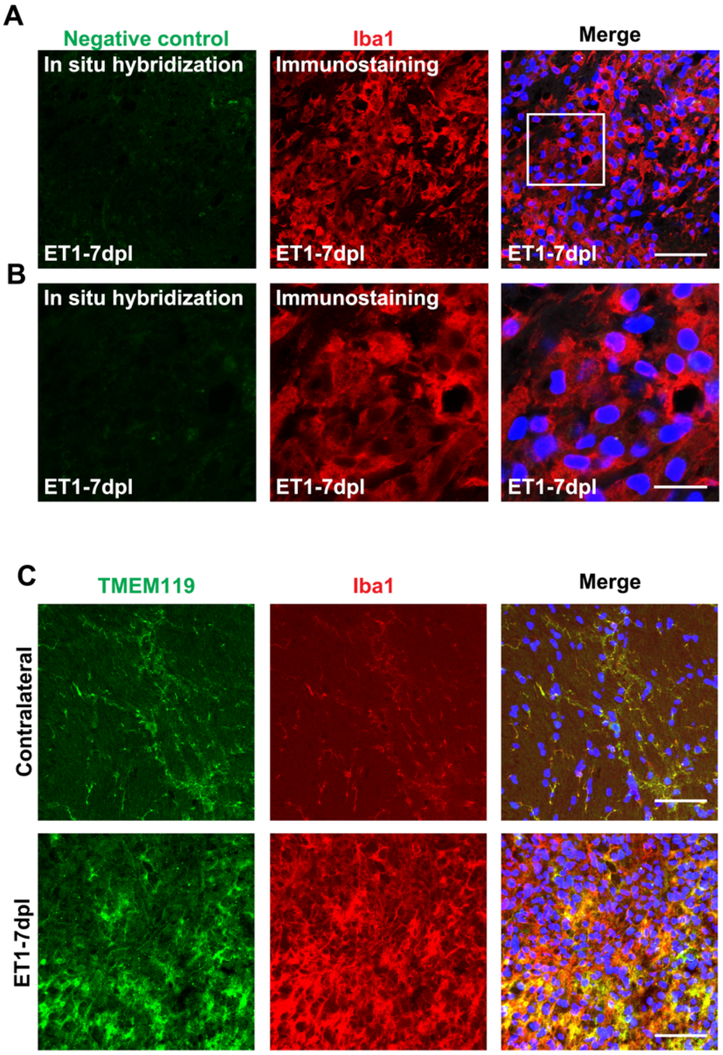

Supplementary Fig. S2 ISH signals of *Col1a1* mRNA (blue) are not present in negative controls. (A) Immunohistochemistry for *Col1a1* mRNA after ISH in the ET1-lesioned IC at 7 dpl without the Col1a1 probe, which was used as a negative control. ISH signals of *Col1a1* mRNA (blue) are not present in the absence of the *Col1a1* probe, but Iba1-positive signals were strongly detected in the lesion at 7 dpl. Scale bar, 50 µm. (B) Enlarged images of the boxed area in (A). Scale bar, 50 µm. (C) Double immunofluorescence images of the contralateral IC and ET1-lesioned IC at 7 dpl labelled with anti-TMEM119 (green) and anti-Iba1 (red) antibodies. Scale bar, 50 µm.

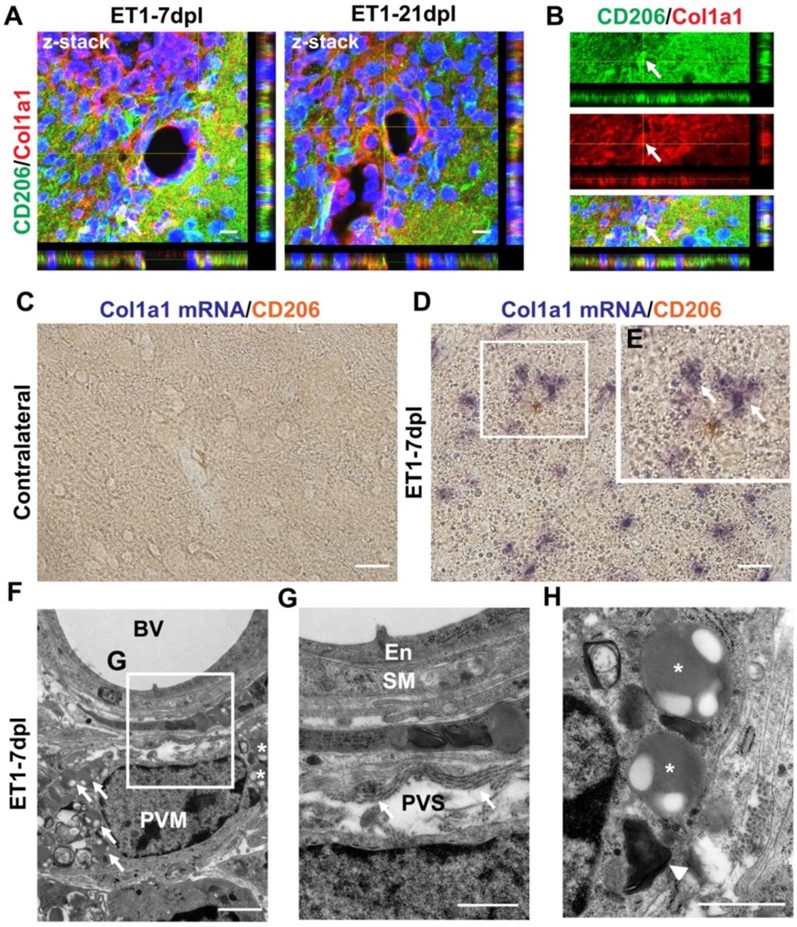

Supplementary Fig. S3 Collagen fibres are secreted by CD206-positive macrophages. (A, B) Z-stack images of double immunofluorescence staining with anti-CD206 (green) and anti-Col1a1 (red) antibodies at 7 and 21 dpl. Colocalization between CD206 and Col1a1 was observed 7 dpl after ET1 injection. (C-E) Immunohistochemistry for *Col1a1* mRNA after ISH in the contralateral IC or ET1-lesioned IC at 7 dpl. Scale bars, 20 μm. (E) Enlarged image of the boxed area in (D). ISH signals of *Col1a1* mRNA (blue) are colocalised with CD206 (brown) at 7 dpl (white arrow). (F) The TEM image shows collagen fibres around the perivascular macrophages (PVM), which contain many lysosomes (white arrows) at 7 dpl. Scale bar, 2 μm. (G) Enlarged image of the boxed area in (F). Scale bar, 1 μm. Collagen fibres (white arrow) were secreted into the perivascular space (PVS). Endothelial cells (En) and smooth muscle (SM) were observed around blood vessels. (H) Asterisks indicate the lysosomes in (F). High electron density lysosomes were also observed (white arrowhead). Scale bar, 1 μm.

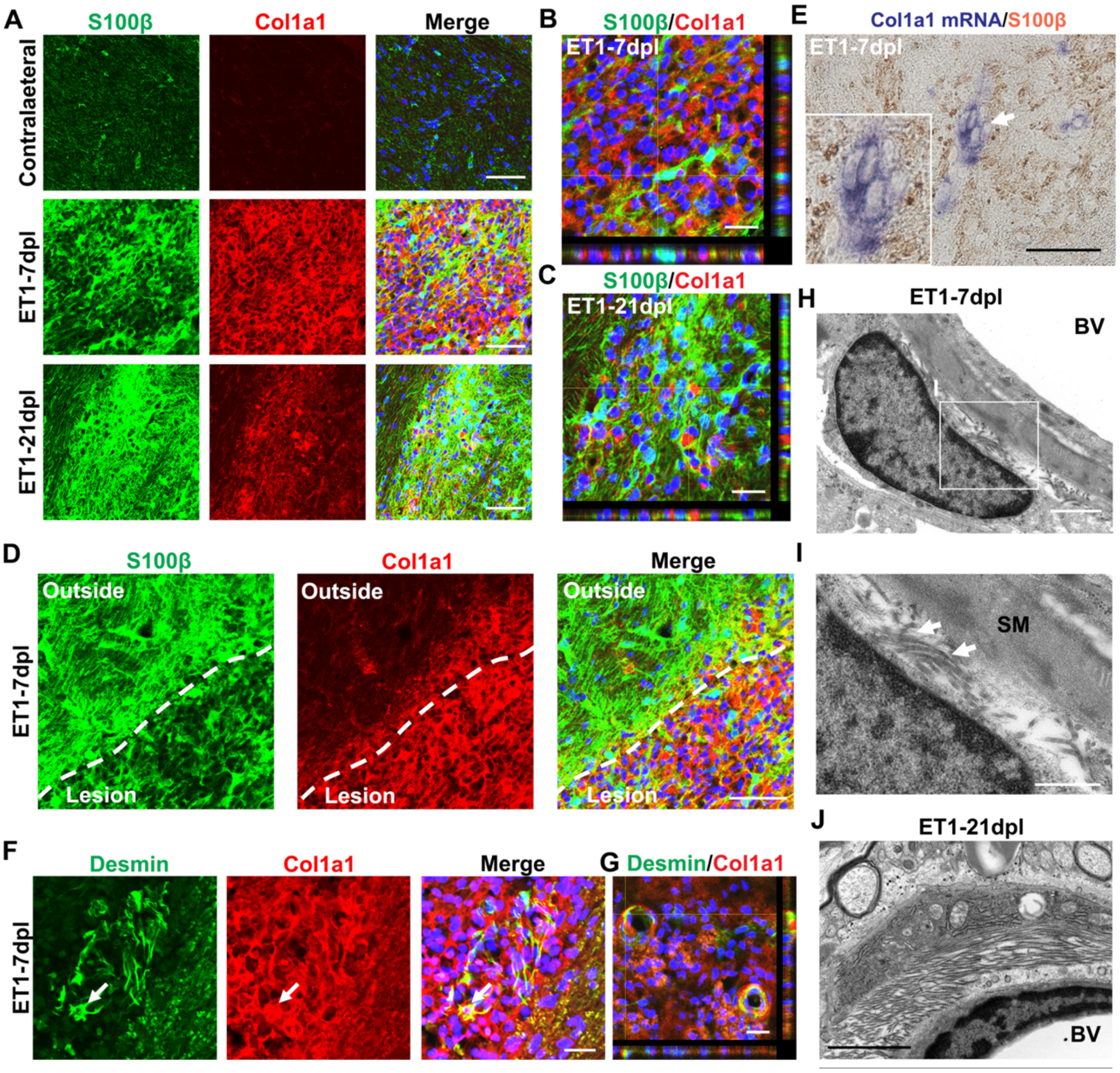

Supplementary Fig. S4 Astrocytes accumulate in perilesional areas in the acute phase after ET1 injection, and perivascular cells produce collagen fibres around blood vessels. (A) Double immunofluorescence images of the ET1-lesioned IC labelled with anti-S100β (green) and anti-Col1a1 (red) antibodies at 7 and 21 dpl. Scale bar, 50 μm. (B, C) Z-stack images of double immunofluorescence staining with anti-Iba1 (green) and anti-Col1a1 (red) antibodies at 7 and 21 dpl. Scale bars, 20 μm. (D) Double immunofluorescence images of the ET1-lesioned IC at 7 dpl labelled with anti-S100β (green) and anti-Col1a1 (red) antibodies. The border of the lesion in ET1-7 dpl is indicated by a white dotted line. Scale bar, 50 μm. (E) Immunohistochemistry for *Col1a1* mRNA after ISH in the contralateral IC or ET1-lesioned IC at 7 dpl. ISH signals of *Col1a1* mRNA (blue) are not colocalised with S100β (brown) at 7 dpl. Scale bar, 20 μm. The white arrow indicates the area of the enlarged image in (E). (F) Double immunofluorescence images of the ET1-lesioned IC at 7 and 21 dpl labelled with anti-desmin (green) and anti-Col1a1 (red)

antibodies. Scale bar, 20 μm. (G) Z-stack images of double immunofluorescence staining at 7 dpl labelled with anti-desmin (green) and anti-Col1a1 (red) antibodies. Scale bar, 20 μm. Nuclei are counterstained with Hoechst (blue). (H-J) TEM analysis shows collagen fibres present around perivascular cells at 7 dpl (H, I) and 21 dpl (J). BV, blood vessel. Scale bars: (H) 1 μm; (I) 500 μm; (J) 2 μm. (I) Enlarged image of the boxed area in (H). Collagen fibres are indicated by the white arrows between the smooth muscle (SM) and perivascular cell.

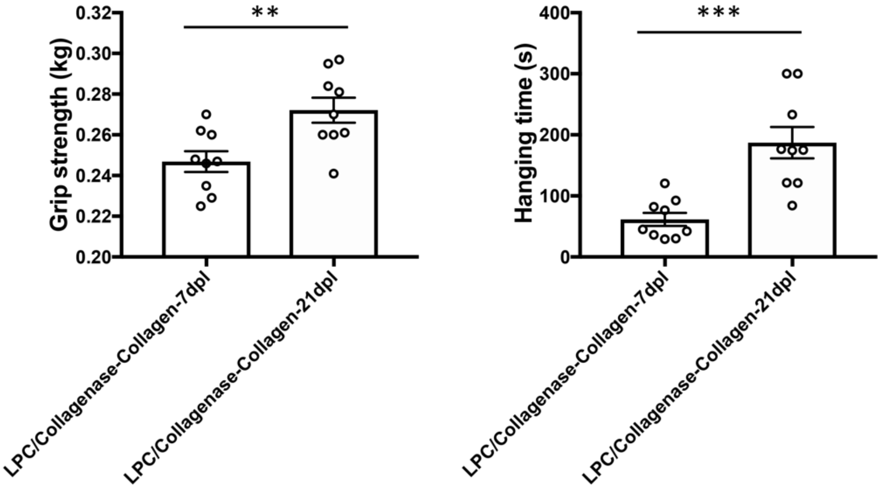

Supplementary Fig. S5 The average grip strength (LPC/Collagenase-Collagen-7dpl, 0.2469 ± 0.005106; LPC/Collagenase-Collagen-21dpl, 0.2721 ± 0.006183) and hanging time (LPC/Collagenase-Collagen-7dpl, 61.5 ± 10.83; LPC/Collagenase-Collagen-21dpl, 187.3 ± 25.64) of LPC/Collagenase-Collagen injected mice at 7 and 21dpl (*n*=9). The mean ± SEM is shown as bars and lines. $^{**}P<0.01$, $^{***}P<0.001$ by Student's *t*-test.

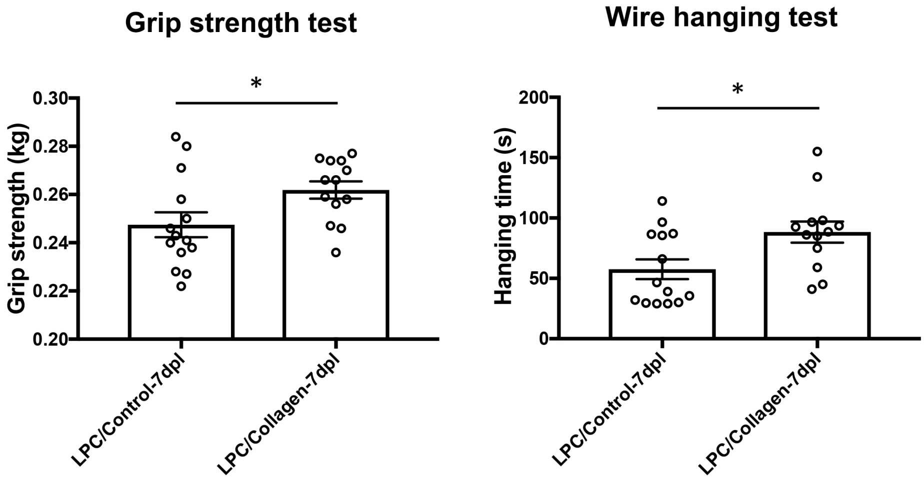

Supplementary Fig. S6 Comparison of the average grip strength (LPC/Control-7dpl, 0.2474 ± 0.005163; LPC/Collagen-7dpl, 0.2618 ± 0.003577) and hanging time (LPC/Control-7dpl, 57.57 ± 8.134; LPC/Collagen-7dpl, 88.38 ± 8.737) between LPC/Collagen and LPC/Control groups (LPC/Control, *n*=14; LPC/Collagen, *n*=13). The mean ± SEM is shown as bars and lines. $^{*}P<0.05$ by Student's *t*-test.

Quantitative values

Behavioural scores for Fig. 2B, C

Fig. 2B, C	Grip strength (kg)	Hanging time (s)
ET1-Pre	0.2791 ± 0.005055	220.50 ± 24.34
ET1-7 dpl	0.2454 ± 0.004456	59.38 ± 14.76
ET1-21 dpl	0.2600 ± 0.004301	97.19 ± 28.8

Graph shows the mean ± standard error of the mean (SEM) obtained from each group (ET1-Pre, *n*=8; ET1-7 dpl, *n*=8; ET1-21 dpl, *n*=8). $^{*}P<0.05$, $^{**}P<0.01$, $^{***}P<0.001$ by Tukey-Kramer test after one-way ANOVA.

Fluorescence intensity for Fig. 2E (left panel)

Fig. 2 E (left panel)	Col1a1 intensity
Lesion	54.4 ± 2.356
Myelinated area	42.5 ± 2.548

Graph shows the mean ± standard error of the mean (SEM) obtained from each group (*n*=3). $^{*}P<0.05$ by Student's *t*-test.

Fluorescence intensity for Fig. 2E (right panel)

Fig. 2 E (right panel)	MBP/Col1a1 intensity
Contralateral	4.166 ± 0.5716
ET1-21 dpl	0.4665 ± 0.03472

Graph shows the mean ± standard error of the mean (SEM) obtained from each group (Contralateral, n=3; ET1-7 dpl, *n*=3). $^{**}P<0.01$ by Student's *t*-test.

Fluorescence intensity for Fig. 3D, E

Fig. 3D, E	Col1a1 intensity	Iba1 intensity
Contralateral	43.98 ± 4.832	29.68 ± 2.207
ET1-7 dpl	171.1 ± 14.41	171.1 ± 1.95
ET1-21 dpl	124.9 ± 8.801	122.4 ± 13.25

Graph shows the mean ± standard error of the mean (SEM) obtained from each group (Contralateral, n=3; ET1-7 dpl, *n*=3; ET1-21 dpl, *n*=4). $^{*}P<0.05$, $^{***}P<0.001$ by Tukey-Kramer test after one-way ANOVA.

Behavioural scores for Fig. 6B

Grip strength (kg) (Fig. 6B)	LPC/Control	LPC/Collagen
7 dpl	0.2474 ± 0.005163	0.2618 ± 0.003577
21 dpl	0.2762 ± 0.004953	0.247 ± 0.007271
**Hanging time (s) (Fig. 6B)**	**LPC/Control**	**LPC/Collagen**
7 dpl	57.57 ± 8.134	88.38 ± 8.737
21 dpl	199.7 ± 21.49	110.0 ± 22.11

Graph shows the mean ± standard error of the mean (SEM) obtained from each group (LPC/Control, *n*=14; LPC/Collagen, *n*=13). $^{*}P<0.05$, $^{**}P<0.01$, $^{***}P<0.001$ by Tukey-Kramer test after one-way ANOVA.

Quantitative analysis for Fig. 6D

Col1a1 intensity (Fig. 8D)	LPC/Control	LPC/Collagen
21dpl	121.6 ± 3.025	151 ± 12.86

Graph shows the mean ± standard error of the mean (SEM) obtained from each group (LPC/Control-21 dpl, *n*=3; LPC/Collagen-21 dpl, *n*=3).

Quantitative analysis for Fig. 6F, G, K

**Iba1 intensity (Fig.6F)**	**LPC/Control**	**LPC/Collagen**
21dpl	30.67 ± 2.162	39.1 ± 1.868
**GFAP intensity (Fig. 6G)**	**LPC/Control**	**LPC/Collagen**
21dpl	36.72 ± 3.976	57.56 ± 5.677
**SMI32 intensity/NF intensity (Fig. 6K)**	**LPC/Control**	**LPC/Collagen**
21 dpl	0.5967 ± 0.05112	0.8595 ± 0.07693

Graph shows the mean ± standard error of the mean (SEM) obtained from each group (LPC/Control-21 dpl, n=5; LPC/Collagen-21 dpl, n=4). $^{*}P$<0.05 by Student's t-test.

Quantitative analysis for Fig. 7G, H

**Olig2^{+} PDGFRA^{+} cells/mm^{2} (lesion) (Fig. 7G)**	**LPC/Control**	**LPC/Collagen**
21 dpl	347.7 ± 30.55	455.1 ± 26.4
**Olig2^{+} CC1^{+} cells/mm^{2} (lesion) (Fig. 7H)**	**LPC/Control**	**LPC/Collagen**
21 dpl	652.9.1 ± 65.26	397.4 ± 19.83

Graph shows the mean ± standard error of the mean (SEM) obtained from each group (LPC/Control-21 dpl, n=4; LPC/Collagen-21 dpl, n=4). $^{*}P$<0.05, $^{**}P$<0.01 by Student's t-test.

Quantitative analysis for Fig. 7J

**MBP intensity (Fig. 7J)**	**LPC/Control**	**LPC/Collagen**
21 dpl	185.6 ± 7.061	128.2 ± 18.19

Graph shows the mean ± standard error of the mean (SEM) obtained from each group (LPC/Control-21 dpl, n=3; LPC/Collagen-21 dpl, n=3). $^{*}P$<0.05 by the Student's t-test.

Quantitative analysis for Fig. 7M

**Myelinated axons/100µm^{2} (Fig. 7M)**	**LPC/Control**	**LPC/Collagen**
21 dpl	7.784 ± 0.7227	12.05 ± 1.161

Graph shows the mean ± standard error of the mean (SEM) obtained from each group (LPC/Control-21 dpl, n=15; LPC/Collagen-21 dpl, n=15). $^{**}P$<0.01 by Student's t-test.

Quantitative analysis for Fig. 7N

**G-ratio (Fig. 7N)**	**LPC/Control**	**LPC/Collagen**
21 dpl	0.7173 ± 0.003079	0.732 ± 0.00373

Graph shows the mean ± standard error of the mean (SEM) obtained from each group (LPC/Control-21 dpl, n=268; LPC/Collagen-21 dpl, n=244). $^{**}P$<0.01 by Student's t-test.
